# Supplementary material for: Complete transition from chromosomal to cytoplasmic sex determination during prolonged Wolbachia symbiosis
Source: Nat Commun. 2026 Jan 8;17:104. doi: 10.1038/s41467-025-67993-x (PMC12783822; doi:10.1038/s41467-025-67993-x)
Supplement: Supplementary file 1 — Supplementary Information [file 41467_2025_67993_MOESM1_ESM.pdf]

Fig. S1

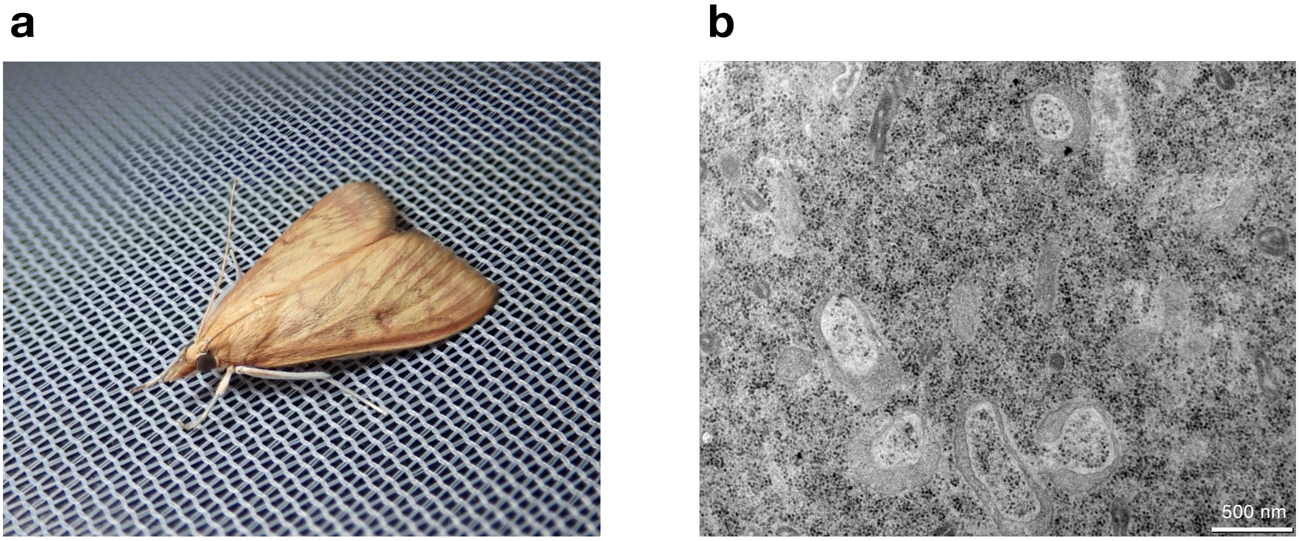

Fig. S1. Male-killing *Wolbachia* in *Ostrinia furnacalis*. (**a**) An adult *O. furnacalis* moth infected with male-killing *Wolbachia w*Fur. (**b**) Transmission electron microscopy image showing *Wolbachia w*Fur within the adult ovary of *O. furnacalis*. The bacteria are enclosed by two host-derived membranes and one *Wolbachia*-derived membrane. Scale bar: 500 nm. Source data are available in the source data file.

Fig. S2

a

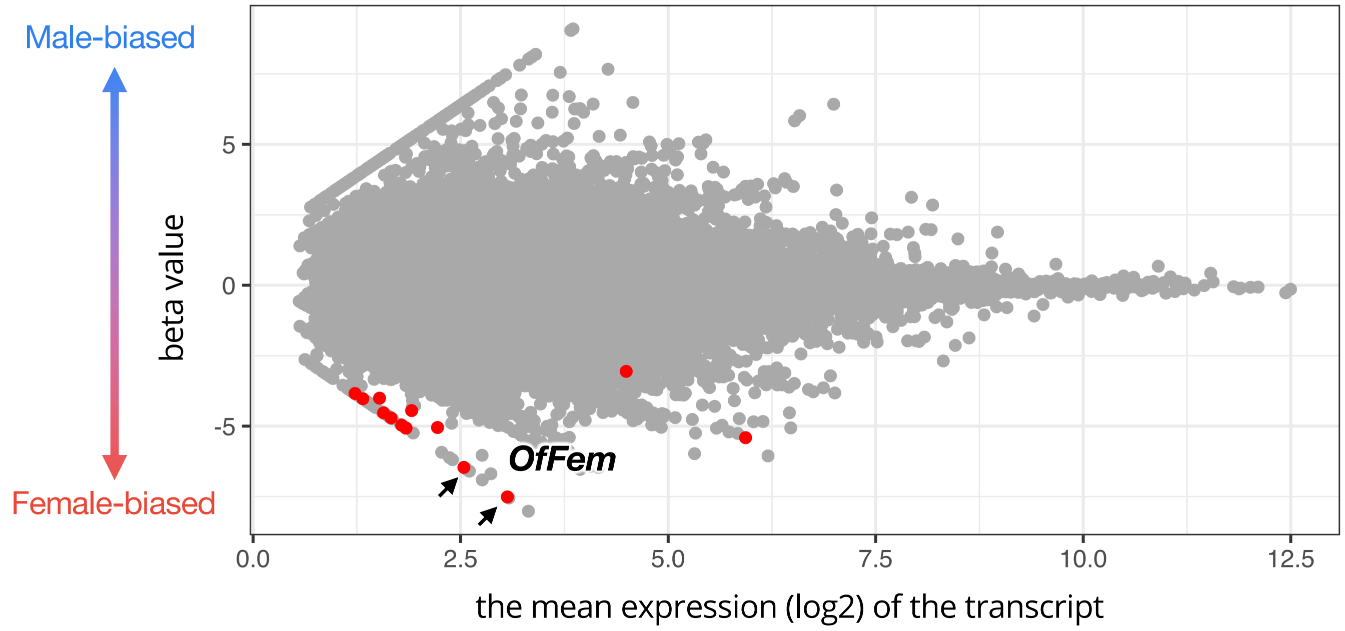
Male-biased
Female-biased
beta value
5
0
-5
OfFem
0.0
2.5
5.0
7.5
10.0
12.5
the mean expression (log2) of the transcript

b

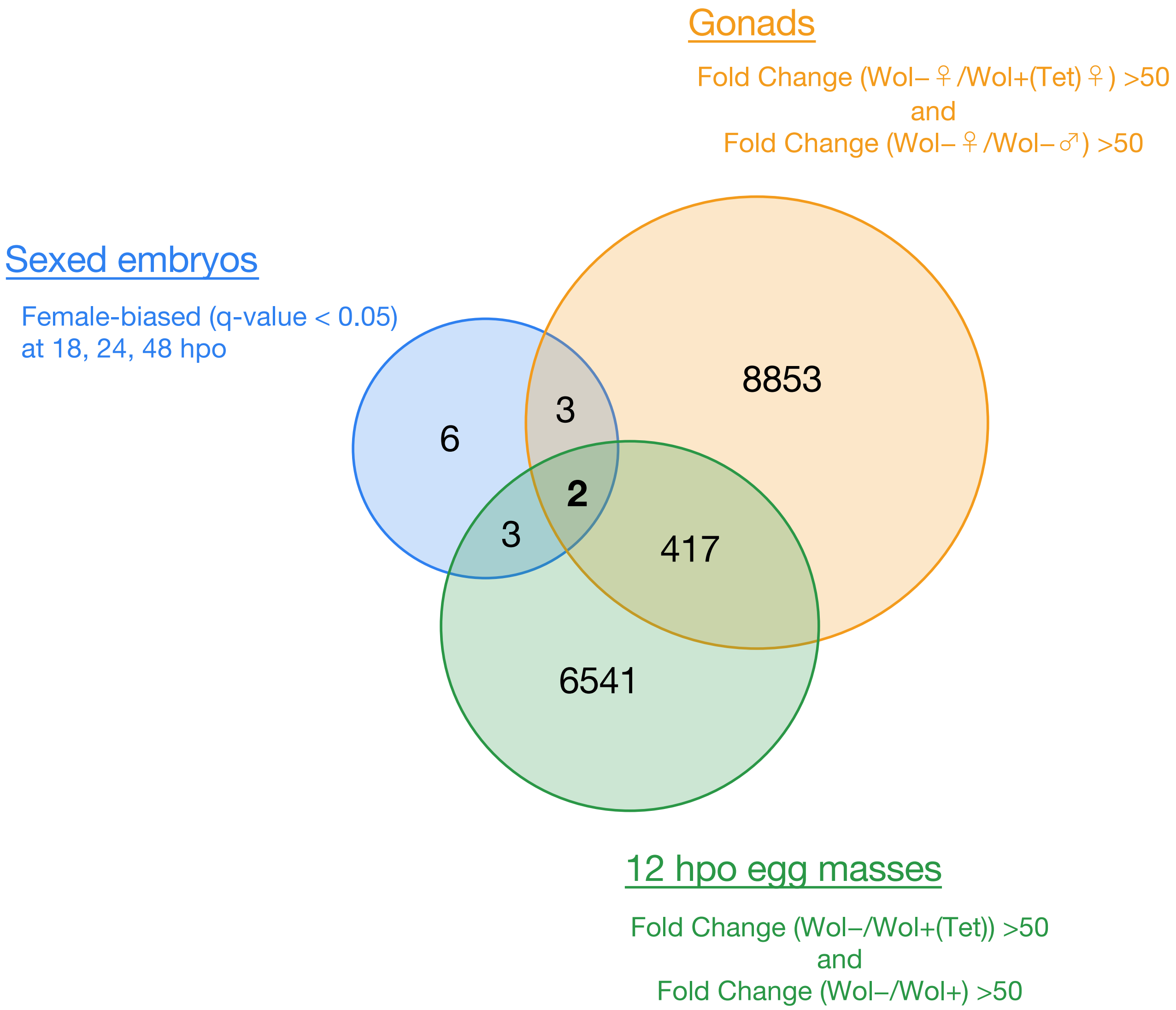
Gonads
Fold Change (Wol−♀/Wol+(Tet)♀) >50
and
Fold Change (Wol−♀/Wol−♂) >50
Sexed embryos
Female-biased (q-value < 0.05)
at 18, 24, 48 hpo
8853
6
3
2
3
417
6541
12 hpo egg masses
Fold Change (Wol−/Wol+(Tet)) >50
and
Fold Change (Wol−/Wol+) >50

Fig. S2. Identification of female-specific transcripts at the embryonic stage in *Ostrinia furnacalis.* (**a**) MA plots showing RNA-seq data from sexed embryos of *O. furnacalis* at 48 hpo. Each dot represents a contig in the transcriptome assembly. The x-axis indicates the log2-mean transcript expression, and the *y*-axis shows the beta value (log2-fold change between sexes). Red dots highlight female-biased contigs with *q*-values <0.05. Two arrows indicate the *OfFem* contigs. (**b**) Venn diagram showing unique and shared contigs meeting various criteria for sex-biased expression based on RNA-seq data. Wol−, *Wolbachia*-uninfected; Wol+, *Wolbachia*-infected; Wol+ (Tet) tetracycline-treated *Wolbachia*-infected. Source data are available in the source data file.

Fig. S3

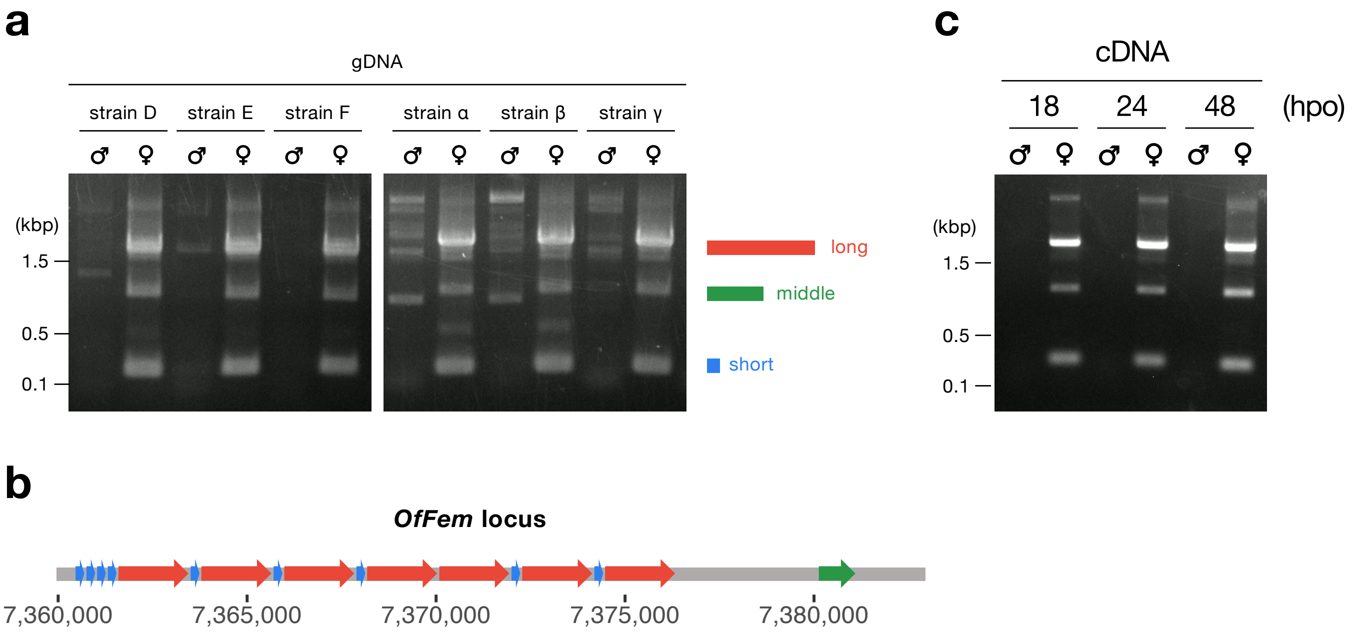


```
>long_fragment (1850 bp)
CTCCACCTCCTGCTTCAGCGTGAGCGGGCACGCGGGAGGCGAGTCCGGAGGCAGCGGCGGTTGCACATATGATTGATAACACTTGTGGTAT
TTTGTTGGCTGTGACTAATGTCACTTAATTCTTTTGGCACTTCATGATGTGTAGATTTTTTAAGGATTCCCGCATAAGTCTCGTTATCTGA
TTTGTTTGACGAGATTTTTGGTAATGGTGCATGGTCTACTGTAGTTCTTGAGTCAGTTCCAGTGTCAATGTGAGTCACGGCGTTTGTCTGT
CTTTGGTGACCGCCTCGTTTGGTTTTTCTGGCTAGAATTTCTTTATAGACCTGGCAGCCCTTGTAGTTAGCTGGGTGATCACTAGAACACA
GGGCACATTTTGCTGGCGAGTTCCTATCTTTTTTGGAGCAGTCAGATGTTTTGTGCCCCTCTCCACATTTGACGCAACGATACGGTCTCAT
GCAGTTGATTTTGGAGTGGCCGTACTGTTGACATCGTTGGCACTGGACAATAGATTTAGACTTACGAGGGTCTTCTATTTTAACTCGTTGT
TGGAAGATATATTGGATGTCTTTGATGGCTTTATTGTTGACATTGGGTTCCAGGTTCACGAAAAATGTGTACGAGGGTAGCTTGTCGGCAC
CAAATCGTGCGTTAATAATCTCGCCACGAACCTTGTTCCCCGTCTTTTCTATTTCTTCTACTATTGCGTCGTGAGGTGTCGTATGGTGTAG
ATTTTTGATGACAATTCGATAGGGTTTAGTGTCCTTTCTAGTAAAGGTGTGTCCTATTAGTCCTTTCTGCCTGACTAAGTCAATCACTTTT
TTGTAATTATCAGCTGAGTTCACCATCAGGCGCAGTAGATTTTTATTCACACTTTTGAATTTAAACTCTGTGGTGGGAATTACTGATTCAA
TCAGTTTTTTGAGCTCATTGACATTCTCGATTCCATATAAGACGATGGGTGGTGGTTTATTGGGTCGATTAGCACTATAAGTTGTATCTTC
TTGATCAAGCGGTAGACCAGTGAATTTATTACCCGTCGGTGTGGGTGAGGGCAAAGGCGGTGAGTCGTCACGTATTTTTCGCTTTTTATTT
GTCGGCACACGTTGCCATTCCGGAGGATGCTGGTTCTGTTCCCCAGGATTAGTGGAGAAGGTTGGAGTGCTGGGTGCTTCCGGGCCGGCTG
CCGTCGTTTGCTCATGTCCGTATTCAGTGCTTCCGCCAAAGGAAGAAGATCTCGGTCGAGAATCGAGGATGCCCTTGTGAAAAACTTGTTG
TACGTAGATTTTTTGATTTTTATTTAGTTTTTTAGTAGTGCTTCCAGAAGCATTCACTATCACGGGAGCCTGGTCGCCCACTCTCGCGCAG
TCTTCGGTAACATATTTCCCACTCGCTGGCCCGGGTACCAACGAGCGCCGGGGGTTAGCCGGATCGCATGACATATCAACATTCGTATCGC
TCATATTTCCATATTTAAGAGTGGGATTCACACATGTCGCTTTCCCAAGGCGACATGGCCACACTTTTAGGGGGGATTTAGTCCTCCTTGG
CTTGTGGTTCACCAGTGGTCAGGTTAGTTAACCGCCGCCTGTTGCTCGGCGTTGTTGCCCATTTGGCACCACCCGGGGGGCACGTGTAGGG
TGGGCCTAGGAGAAGGTGGTAAGTCCTACGGACGCGAGCAACTATACCCCCCAACCACGGAAATGACTTTATTTTTTCTTTTGACTTCGTT
CGCAATCGCTTCGGAGTTATGAATTTAATCTTCTTCTTGGAAGTATTTTTTCATGTGTTTTTGACACCGCTCCATCCTAAATTCTGAGCTG
GCCCTTGTCGAGCTCGTGCGCGTAGATGCA
>middle_fragment (969 bp)
CTCCACCTCCTGCTTCAGCGTGAGCGGGCACGCGGGAGGCGAGTCCGGAGGCAGCGGCGGTTGCACCAGGGCCGTAGCTAGGGTCGAATTT
GAGGTAGGGCAGCATTAGTTACAGGTAGGGCGGAAGTAAAAAAAATATCATAATTGATCTGCCGTCGAAACACGGGCTCCTAAATCAATCA
CCATGTATATCACCATCATGTGAATAGTTATTCAAATAATTACTGTGACAGTGCATGAGCATGGATCTAAGAACTATTAAATCCTCAATGA
TGAAGAACAAGTGAATAAGATGGTTGAACTTCCAGATTTGGATGTAGAGATCCACGTCTGAGTGAGAGTGAGGTAATAATTAATTATTGGG
TGGGGTGATACGAGTAAATAGAAACGACCAAAAATATCATTAAGCGCTTCGAATGCCATCTATACACAGAACAAAGAATGTAACACTTTTT
TTTGCATTCACTGATAAAACACCTGAAATTCAATAAAATATAAACAATATCCATTTGAGGTTGTTTTTGTTTTGACGTTTTGTTTTTATTC
AGCAAAATGTCATCATGACATTTGTCATTGGTGCGTTCCGTTTCATCGGCTTTAGTTTGAACCAATACAAGAGTCTTACCATCACATTATA
AATTTTGTTGCAAACTTAGATACCTATCGCTACATTTTTTTTCTCAAACTATGAACAAACAATTTAATTATAATAATTTATCTTAGTTTTA
GATATTTTTAAGGTAGGGCATTGTGGTTTCAAGGTAGGGCATTGCCCCACAATGCCCCCCCCTAGCTACGGCCATGGGTTGCACCACGGAA
ATGACTTTATTTTTTCTTTTGACTTCGTTCGCAATCGCTTCGGAGTTATGAATTTAATCTTCTTCTTGGAAGTATTTTTTCATGTGTTTTT
GACACCGCTCCATCCTAAATTCTGAGCTGGCCCTTGTCGAGCTCGTGCGCGTAGATGCA
>short_fragment (223 bp)
CTCCACCTCCTGCTTCAGCGTGAGCGGGCACGCGGGAGGCGAGTCCGGAGGCAGCGGCGGTTGCACCACGGAAATGACTTTATTTTTTCTT
TTGACTTCGTTCGCAATCGCTTCGGAGTTATGAATTTAATCTTCTTCTTGGAAGTATTTTTTCATGTGTTTTTGACACCGCTCCATCCTAA
ATTCTGAGCTGGCCCTTGTCGAGCTCGTGCGCGTAGATGCA
```

Fig. S3. Female-specific detection and expression of the *OfFem* locus in *Ostrinia furnacalis*. (**a**) Detection of the *OfFem* locus from genomic DNAs in *O. furnacalis*. Strains D, E, F and strains α, β, γ were established from different founder moths and separately maintained. Multiple non-specific bands were amplified from the male sample of strains α, β, and γ. (**b**) Mapping of PCR-amplified fragments (from panel A) onto the *OfFem* locus (top). The nucleotide sequences of these PCR-amplified fragments are also shown (bottom). **(c)** Female-specific expression of the *OfFem* locus from embryonic cDNA as detected by RT-PCR. Source data are available in the source data file.

Fig. S4

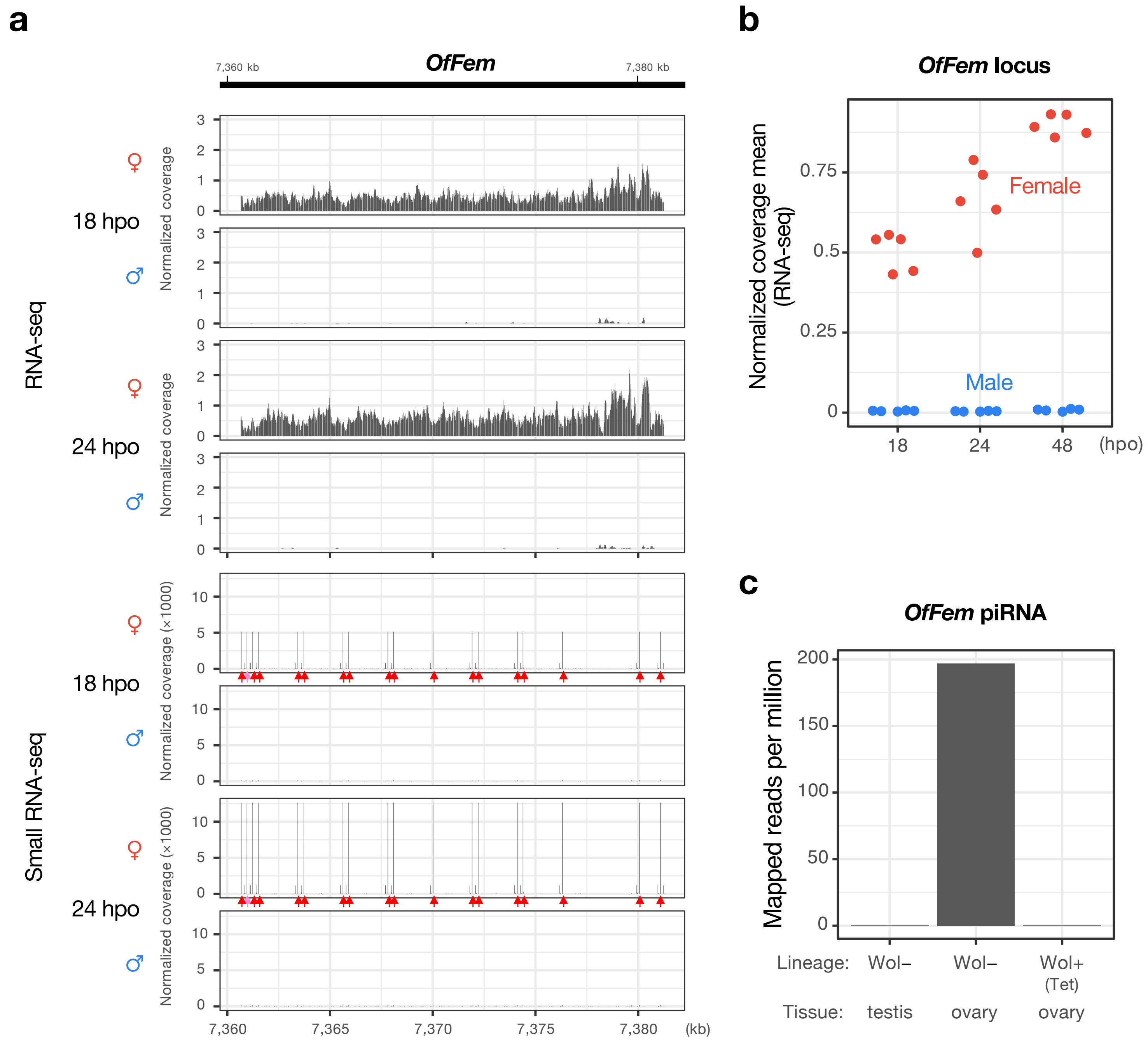

Fig. S4. Expression profile of the *OfFem* locus and *OfFem* piRNA in *Ostrinia furnacalis*. (**a**) Mapping of RNA-seq and small RNA-seq reads from sexed embryos at 18 and 24 hpo onto the *OfFem* locus. Arrows indicate the *OfFem* piRNA-production sites. Red arrows denote exact matches to *OfFem* piRNA, and pink arrows show sequences with a single nucleotide mismatch. (**b**) Expression profile of the *OfFem* locus in individual sexed embryos, with red dots indicating female (n = 5) values and blue dots male (n = 5) values. (**c**) Expression levels of *OfFem* piRNA in the testis and ovary from uninfected lineage (Wol−) and in the ovary from tetracycline-treated moths from the *Wolbachia*-infected lineage (Wol+ (Tet)) (n = 1). Source data are available in the source data file.

Fig. S5

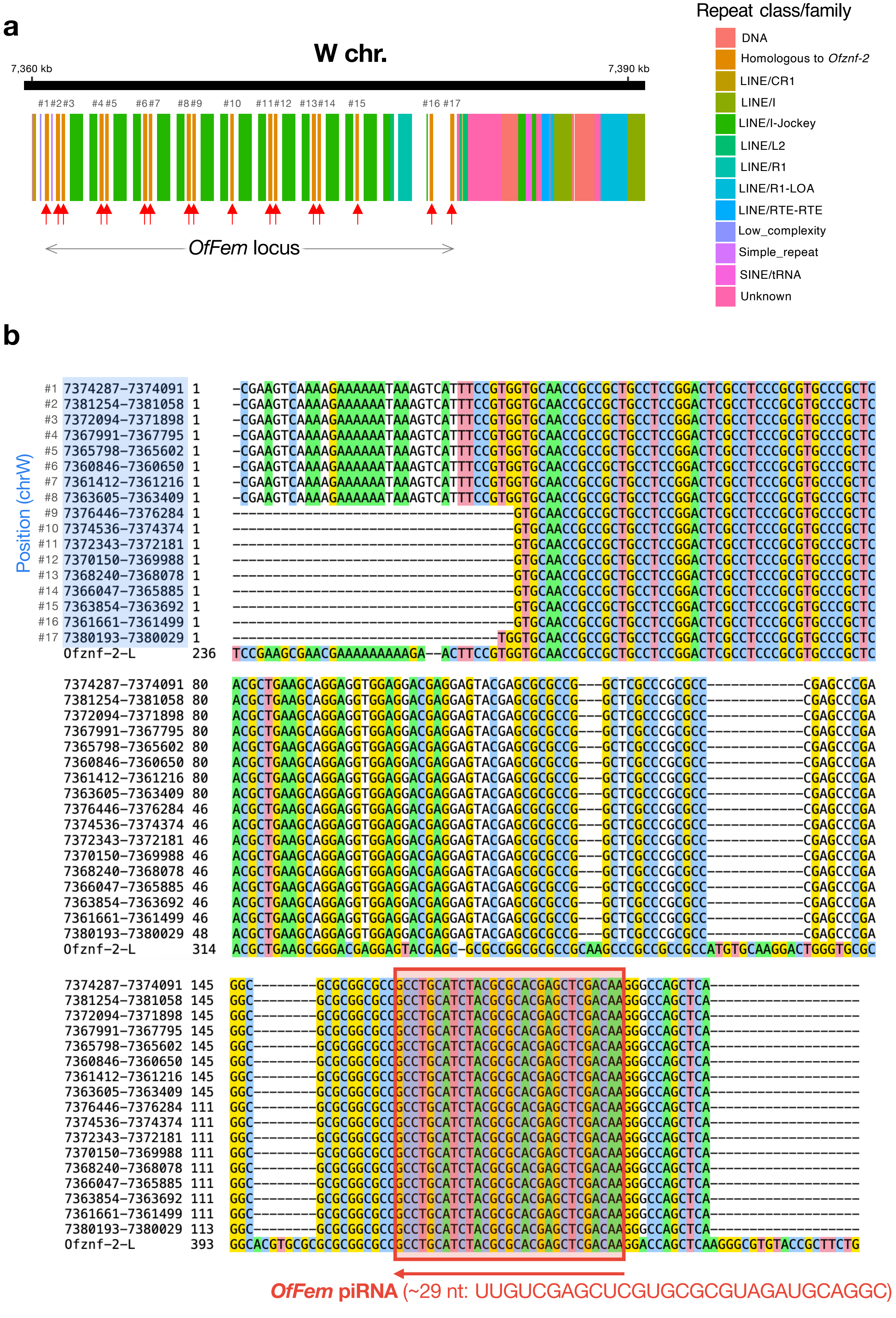

a
W chr.
7,360 kb
7,390 kb
#1#2#3 #4#5 #6#7 #8#9 #10 #11#12 #13#14 #15 #16 #17
OfFem locus
Repeat class/family
DNA
Homologous to Ofznf-2
LINE/CR1
LINE/I
LINE/I-Jockey
LINE/L2
LINE/R1
LINE/R1-LOA
LINE/RTE-RTE
Low_complexity
Simple_repeat
SINE/tRNA
Unknown
b
Position (chrW)
#1 7374287-7374091
#2 7381254-7381058
#3 7372094-7371898
#4 7367991-7367795
#5 7365798-7365602
#6 7360846-7360650
#7 7361412-7361216
#8 7363605-7363409
#9 7376446-7376284
#10 7374536-7374374
#11 7372343-7372181
#12 7370150-7369988
#13 7368240-7368078
#14 7366047-7365885
#15 7363854-7363692
#16 7361661-7361499
#17 7380193-7380029
Ofznf-2-L
OfFem piRNA (~29 nt: UUGUCGAGCUCGUGCGCGUAGAUGCAGGC)

Fig. S5. Sequence analysis of the *OfFem* locus in *Ostrinia furnacalis*. (**a**) Genomic organization of the *OfFem* locus, highlighting the transposons and repeat regions. Red arrows indicate the positions of the *OfFem* piRNA sequences within the locus. (**b**) Nucleotide alignment of *Ofznf-2-L* and repeat sequences homologous to *Ofznf-2* within the *OfFem* locus, showing regions of sequence conservation.

Fig. S6

```
Ofznf-2-L            ------------------------------------------------------------
Ofznf-2-E(chr28#2)   ------------------------------------------------------------
Ofznf-2-E(chr28#1)   ------------------------------------------------------------
Ofznf-2-E(chrZ)      ATGAAAAAATATCTTAAGTCCAAATTTGAAGAAGATCAAATTCAAATATCCGAAGCGAAC
Ofznf-2-W1,W2        ATGAAAAAATATCTTAAGTCCAAATTTGAAGAAGATCAAATTCAAATATCCGAAGCGAAC

Ofznf-2-L            ------------------------------------------------------------
Ofznf-2-E(chr28#2)   ------------------------------------------------------------
Ofznf-2-E(chr28#1)   ------------------------------------------------------------
Ofznf-2-E(chrZ)      GAAAAAAAAAGAACTTCCGTGGTGCAACCGCCGCTGCCTCCGGACTCGCCTCCCGCGTGC
Ofznf-2-W1,W2        GAAAAAAAAAGAACTTCCGTGGTGCAACCGCCGCTGCCTCCGGACTCGCCTCCCGCGTGC

Ofznf-2-L            ------------------------------------------------------------
Ofznf-2-E(chr28#2)   ------------------------------------------------------------
Ofznf-2-E(chr28#1)   ------------------------------------------------------------
Ofznf-2-E(chrZ)      CCGCTCACGCTGAAGCAGGAGGTGGAGGACGAGGAGTACGAGCGCGCCGGCTCGCCCGCG
Ofznf-2-W1,W2        CCGCTCACGCTGAAGCGGGAGGTGGAGGACGAGGAGTACGAGCGCGCCGGCTCGCCCGCG

Ofznf-2-L            ---------------------------------------ATGTGCAAGGACTGGGTGCGC
Ofznf-2-E(chr28#2)   ---------------------------------------ATGTGCAAGGACTGGGTGCGC
Ofznf-2-E(chr28#1)   ---------------------------------------ATGTGCAAGGACTGGGTGCGC
Ofznf-2-E(chrZ)      CCCGAGTCCGAGCCGGCGCGCCGCAAGCCCGCCGCCGCCATGTGCAAGGACTGGGTGCGC
Ofznf-2-W1,W2        CCCGCGCCCGCGCCGGCGCGCCGCAAGCCCGCCGCCGCCATGTGCAAGGATTGGGTGCGC

Ofznf-2-L            GGCACGTGCGCGCGCGGCGCCGCCTGCATCTACGCGCACGAGCTCGACAAGGACCAGCTC
Ofznf-2-E(chr28#2)   GGCACGTGCGCGCGCGGCGCCGCCTGCATCTACGCGCACGAGCTCGACAAGGACCAGCTC
Ofznf-2-E(chr28#1)   GGCACGTGCGCGCGCGGCGCCGCCTGCATCTACGCGCACGAGCTCGACAAGGACCAGCTC
Ofznf-2-E(chrZ)      GGCACGTGCGCGCGCGGCGCCGCCTGCATCTACGCGCACGAGCTCGACAAGGACCAGCTC
Ofznf-2-W1,W2        GGCACGTGCGCGCGCGGCGCCGCCTGCATCTACGCGCACGAGCTCGACAAGGACCAGCTC

Ofznf-2-L            AGGGGCGTGTACCGCTTCTGTCGCGACTTCGAGAACGACCGCTGCGAGCGCCAGGTGTGC
Ofznf-2-E(chr28#2)   AGGGGCGTGTACCGCTTCTGCCGCGACTTCGAGAACGACCGCTGCGAGCGCCAGGTGTGC
Ofznf-2-E(chr28#1)   AGGGGCGTGTACCGCTTCTGCCGCGACTTCGAGAACGACCGCTGCGAGCGCCAGGTGTGC
Ofznf-2-E(chrZ)      AGGGGCGTGTACCGCTTCTGTCGCGACTTCGAGAACGACCGCTGCGAGCGCCAGGTGTGC
Ofznf-2-W1,W2        AAGGGCGTGTACCGCTTCTGCCGCGACTTCGAGAACGACCGCTGCGAGCGCCAGGTGTGC

Ofznf-2-L            TACTTCGTGCACGCCACCACCTTCGAGAAGGAGCACTTCTTCCGCACCGCCTTCCTGCCG
Ofznf-2-E(chr28#2)   TACTTCGTGCACGCCACCACCTTCGAGAAGGAGCACTTCTTCCGCACCGCCTTCCTGCCG
Ofznf-2-E(chr28#1)   TACTTCGTGCACGCCACCACCTTCGAGAAGGAGCACTTCTTCCGCACCGCCTTCCTGCCG
Ofznf-2-E(chrZ)      TACTTCGTGCACGCCACCACCTTCGAGAAGGAGCACTTCTTCCGCACCGCCTACCTGCCG
Ofznf-2-W1,W2        TACTTCGTGCACGCCACCACCTTCGAGAAGGAGCACTTCTTCCGCACCGCCTTCCTGCCG

Ofznf-2-L            CCGCACGCGCTGTACCACCTCAAGACCGCC------------------------------
Ofznf-2-E(chr28#2)   CCGCACGCGCTGCACCACCTCAAGACCG---------------------TCGAAGGTAAC
Ofznf-2-E(chr28#1)   CCGCACGCGCTGCACCACCTCAAGACCG---------------------TCGAAGGTAAC
Ofznf-2-E(chrZ)      CCGCACGCGCTGCACCACCTCAAGACCGCCGCGCCGCAGCCGCCGCCCGTCGAAGGTAAC
Ofznf-2-W1,W2        CCGCACGCGCTGCACCACCTCCAGACCGCCGCGCCGCAGCCGCCGCCCGTCGAAGGTAAC

Ofznf-2-L            ------------------------------------------------------------
Ofznf-2-E(chr28#2)   GCCCGCGGAGTA---------------------------------------CGCGCGGCT
Ofznf-2-E(chr28#1)   GCCCGCGGAGTAGGCGCCGCGCCCGCGCCCTGCGCCCCGCGCCCCGCGCCCCGCGCGGCT
Ofznf-2-E(chrZ)      GCCCGCGGAGTCGGCGCCGCGCCCGCGCCC-GCGCCC-GCGCCCCGCGCCCCGCGCGGCT
Ofznf-2-W1,W2        GCCCGCGGAGTAGGCGCCGCGCCCGCGCCCTGCG-------CCCCGCGCCCCGCGCGGCT

Ofznf-2-L            -------------------------------------------GTGATTGGTCTGTGTAT
Ofznf-2-E(chr28#2)   CTATTTACGGACAATTATCGATATAGTTATTATCTGCGAACTAGTGATTGGTCTGTGTAT
Ofznf-2-E(chr28#1)   CTATTTACGGACAATTATCGATATAGTTATTATCTGCGAACTAGTGATTGGTCTGTGTAT
Ofznf-2-E(chrZ)      CTATTTACGGACAATTATCGATATAGTTATTATCTGCGAACTAGTGATTGGTCTGTGTAT
Ofznf-2-W1,W2        CTATTTACGGACAAT--(6089 bp insertion)--GTGATTGGTCTGTGTAT

Ofznf-2-L            CGTAAGTTTTTCCATTATTGTGGGCCTTTTTCTTTTAAGCAAAACAAGGCAGGTATTTGA
Ofznf-2-E(chr28#2)   CGTAAGTTTTTCCATTATTGTGGGCCTTTTTCTTTTAAGCAAAACAAGGCAGGTATTTGA
Ofznf-2-E(chr28#1)   CGTAAGTTTTTCCATTATTGTGGGCCTTTTTCTTTTAAGCAAAACAAGGCAGGTATTTGA
Ofznf-2-E(chrZ)      ------------------------------------------------------------
Ofznf-2-W1,W2        CGTAAGTTTTTCCATTATTGTGGGCCTTTTTCTTTTAAGCAAAACAAGGCAGGTATTTG

Ofznf-2-L            CCGCAATCACACCTGA
Ofznf-2-E(chr28#2)   ----------------
Ofznf-2-E(chr28#1)   ----------------
Ofznf-2-E(chrZ)      ----------------
Ofznf-2-W1,W2        CCGCAATCACACCTGA
```

UUGUCGAGCUCGUGCGCGUAGAUGCAGGC ← *OfFem* piRNA

Ofznf-2-E_F

Ofznf-2-E_R

E-type-specific insertion

Fig. S6. Presence of five *Ofznf-2* copies in the *Ostrinia furnacalis* genome assembly. The nucleotide alignment of *Ofznf-2* variants found in the version #2 genome assembly is shown. Start and stop codons are marked in blue. The putative *OfFem* piRNA target site in *Ofznf-2* is shaded red. Positions of the *Ofznf-2-E*-specific PCR primers used in Fig. S7a are indicated by green arrows.

Fig. S7

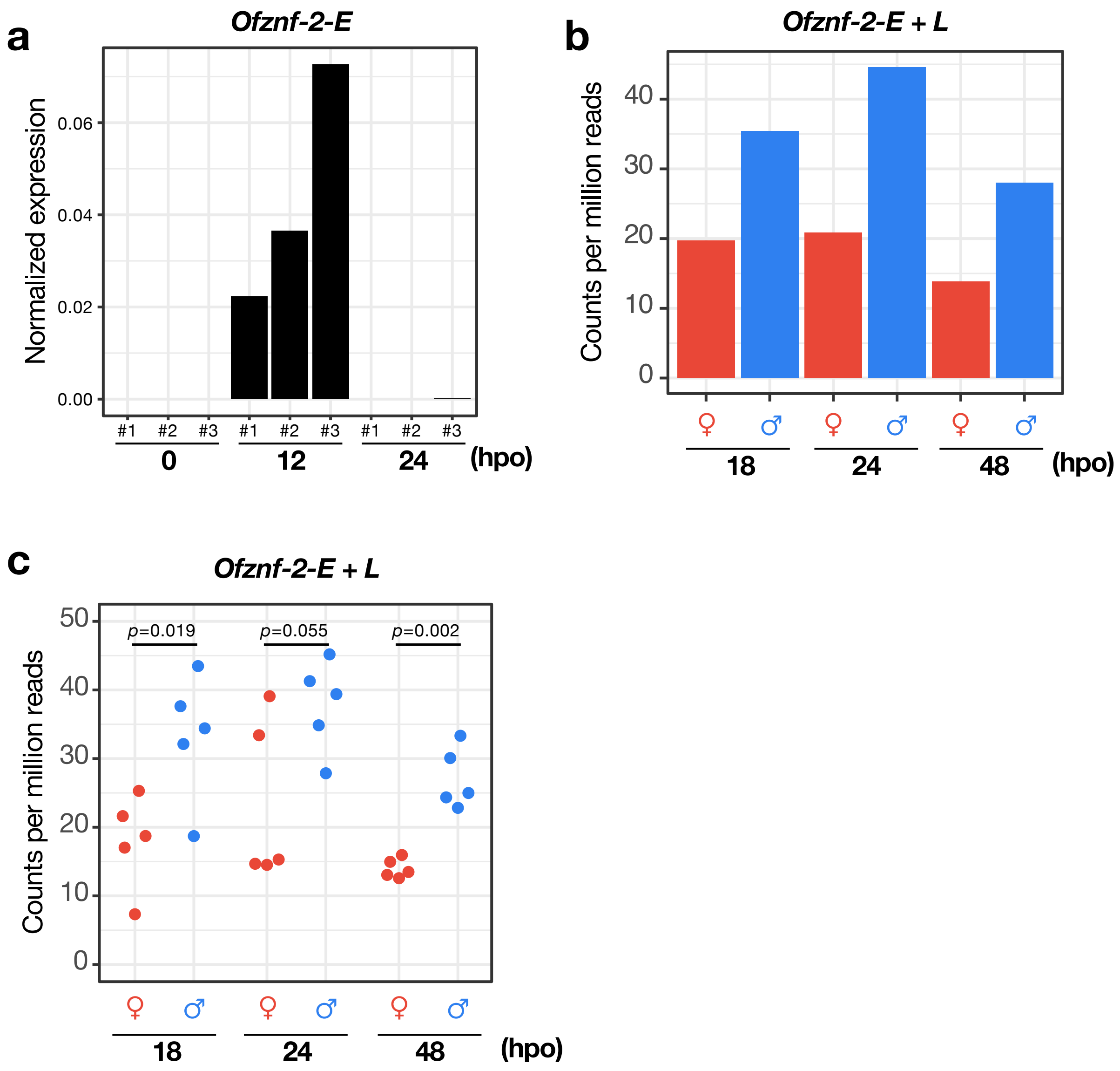

Fig. S7. Expression profile of *Ofznf-2* during the embryonic stage in *Ostrinia furnacalis*. (**a**) Temporal expression of *Ofznf-2-E* analyzed by RT-qPCR in egg masses (n = 3) collected at 0, 12, and 24 hpo. (**b, c**) Expression of *Ofznf-2* from RNA-seq data at 18, 24, and 48 hpo. (b) Data from pooled egg samples (n = 1). (c) Data from single egg samples (n = 5). Statistical analysis was performed using Welch's *t*-test to determine significance at each time point. Source data are available in the source data file.

Fig. S8

```
Dai_etal_E(chr24)     ------------------------------------------------------------
Ver2_E(chr28#1)       ------------------------------------------------------------
Ver2_E(chr28#2)       ------------------------------------------------------------
Ver2_L                ------------------------------------------------------------
Ver1_L                ------------------------------------------------------------
Dai_etal_L            ------------------------------------------------------------
Ver2_E(chrZ)          MKKYLKSKFEEDQIQISEANEKKR----TSVVQPPLPPDSPPACPLTLKQEVEDEEYERA
Ver1_E(chrZ)          MKKYLKSKFEEDQIQISEANEKKR----TSVVQPPLPPDSPPACPLTLKQEVEDEEYERA
Dai_etal_E(chrZ)      MKKYLKSKFEEDQIQISEANEKKRKNKVISVVQPPLPPDSPPACPLTLKQEVEDEEYERA

Dai_etal_E(chr24)     -----------------MCKDWVRGTCARGAACIYAHELDKDQLRGVYRFCRDFENDRCE
Ver2_E(chr28#1)       -----------------MCKDWVRGTCARGAACIYAHELDKDQLRGVYRFCRDFENDRCE
Ver2_E(chr28#2)       -----------------MCKDWVRGTCARGAACIYAHELDKDQLRGVYRFCRDFENDRCE
Ver2_L                -----------------MCKDWVRGTCARGAACIYAHELDKDQLRGVYRFCRDFENDRCE
Ver1_L                -----------------MCKDWVRGTCARGAACIYAHELDKDQLRGVYRFCRDFENDRCE
Dai_etal_L            -----------------MCKDWVRGTCARGAACIYAHELDKDQLRGVYRFCRDFENDRCE
Ver2_E(chrZ)          GSPAPESEPARRKPAAAMCKDWVRGTCARGAACIYAHELDKDQLRGVYRFCRDFENDRCE
Ver1_E(chrZ)          GSPAPESEPARRKPAAAMCKDWVRGTCARGAACIYAHELDKDQLRGVYRFCRDFENDRCE
Dai_etal_E(chrZ)      GSPAPEPEPARRKPAAAMCKDWVRGTCARGAACIYAHELDKSQLKGVYRFCRDFENDRCE
                                       ************************.**:***************

Dai_etal_E(chr24)     RQVCYFVHATTFEKEHFFRTAFLPPHALHHLKTVEGNARRVGAAPAPCAPRPAPRAALFT
Ver2_E(chr28#1)       RQVCYFVHATTFEKEHFFRTAFLPPHALHHLKTVEGNARGVGAAPAPCAPRPAPRAALFT
Ver2_E(chr28#2)       RQVCYFVHATTFEKEHFFRTAFLPPHALHHLKTVEGNAR-------------GVRAALFT
Ver2_L                RQVCYFVHATTFEKEHFFRTAFLPPHALYHLKTAVIGLCIVSFSIIVGLFLLSKTRQVFD
Ver1_L                RQVCYFVHATTFEKEHFFRTAFLPPHALHHLKTAVIGLCIVSFSIIVGLFLLSKTRQVFD
Dai_etal_L            RQVCYFVHATTFEKEHFFRTAFLPPHALHHLKTAVIGLCIVSFSIIVSLFLLSKTRQVFD
Ver2_E(chrZ)          RQVCYFVHATTFEKEHFFRTAYLPPHALHHLKTAAPQPPPVEGNARGVGAAPAPAPAPRA
Ver1_E(chrZ)          RQVCYFVHATTFEKEHFFRTAYLPPHALHHLKTAAPQPPPVEGNARGVGAAPAPAPAPRA
Dai_etal_E(chrZ)      RQVCYFVHATTFEKEHFFRTAYLPPHALHHLKTAAPQPPPVEGNARGVGAAPAPR----A
                      *********************:******:****.                  .

Dai_etal_E(chr24)     DNYRYSYYLRTSDWSVYRKFFHYCGPFSFKQNKAGI
Ver2_E(chr28#1)       DNYRYSYYLRTSDWSVYRKFFHYCGPFSFKQNKAGI    E-type
Ver2_E(chr28#2)       DNYRYSYYLRTSDWSVYRKFFHYCGPFSFKQNKAGI
Ver2_L                RNHT--------------------------------
Ver1_L                RNHT--------------------------------    L-type
Dai_etal_L            RNHA--------------------------------
Ver2_E(chrZ)          PRGSIYGQLSI-------------------------
Ver1_E(chrZ)          PRGSIYGQLSI-------------------------    E-type
Dai_etal_E(chrZ)      PRGSIYGQLSI-------------------------
                       .
```

Fig. S8. Alignment of Ofznf-2-L and Ofznf-2-E protein sequences from three genome assemblies. The alignment shows Ofznf-2 protein sequences derived from genome assembly version #1, version #2, and a published genome assembly as described in reference (*1*). Note that chr 24 in reference (*1*) corresponds to chr 28 in version #1 and version #2 (Table S5).

Fig. S9

a

```
Ofznf-2-L              ------------------------------------------------------------
Ofznf-2-E(chr28#2)     ------------------------------------------------------------
Ofznf-2-E(chr28#1)     ------------------------------------------------------------
Ofznf-2-E(chrZ)        MKKYLKSKFEEDQIQISEANEKKRTSVVQPPLPPDSPPACPLTLKQEVEDEEYERAGSPA
Ofznf-2-E_clone#1      ------------------------------------------------------------
Ofznf-2-E_clone#2      ------------------------------------------------------------
Ofznf-2-E_clone#3      ------------------------------------------------------------

Ofznf-2-L              -------------MCKDWVRGTCARGAACIYAHELDKDQLRGVYRFCRDFENDRCERQVC
Ofznf-2-E(chr28#2)     -------------MCKDWVRGTCARGAACIYAHELDKDQLRGVYRFCRDFENDRCERQVC
Ofznf-2-E(chr28#1)     -------------MCKDWVRGTCARGAACIYAHELDKDQLRGVYRFCRDFENDRCERQVC
Ofznf-2-E(chrZ)        PESEPARRKPAAAMCKDWVRGTCARGAACIYAHELDKDQLRGVYRFCRDFENDRCERQVC
Ofznf-2-E_clone#1      -------------MCKDWVRGTCARGAACIYAHELDKDQLRGVYRFCRDFENDRCERQVC
Ofznf-2-E_clone#2      -------------MCKDWVRGTCARGAACIYAHELDKDQLRGVYRFCRDFENDRCERQVC
Ofznf-2-E_clone#3      -------------MCKDWVRGTCARGAACIYAHELDKDQLRGVYRFCRDFENDRCERQVC

Ofznf-2-L              YFVHATTFEKEHFFRTAFLPPHALYHLKTAVIGLCIVSFSIIVGLFLLSKTRQVFDRNHT
Ofznf-2-E(chr28#2)     YFVHATTFEKEHFFRTAFLPPHALHHLKT-------VEGNARGV--------------RA
Ofznf-2-E(chr28#1)     YFVHATTFEKEHFFRTAFLPPHALHHLKT-------VEGNARGVGAAPAPC-APRPAPRA
Ofznf-2-E(chrZ)        YFVHATTFEKEHFFRTAYLPPHALHHLKTAAPQPPPVEGNARGVGAAPAPAPAPR-APR-
Ofznf-2-E_clone#1      YFVHATTFEKEHFFRTAFLPPHALHHLKT-------VEGNARGVGAAPAPC-APRPAPRA
Ofznf-2-E_clone#2      YFVHATTFEKEHFFRTAFLPPHALHHLKT-------VEGNARGVGAAPAPC-APRPAPRA
Ofznf-2-E_clone#3      YFVHATTFEKEHFFRTAFLPPHALHHLKT-------VEGNARGVGAAPAPC-APRPAPRA

Ofznf-2-L              -----------------------------------------
Ofznf-2-E(chr28#2)     ALFTDNYRYSYYLRTSDWSVYRKFFHYCGPFSFKQNKAGI-
Ofznf-2-E(chr28#1)     ALFTDNYRYSYYLRTSDWSVYRKFFHYCGPFSFKQNKAGI-
Ofznf-2-E(chrZ)        --GSIYGQLSI------------------------------
Ofznf-2-E_clone#1      ALFTDNYRYSYYLRTSDWSVYRKFFHYCGPFSFKQNKAGI-
Ofznf-2-E_clone#2      ALFTDNK-LSI------------------------------
Ofznf-2-E_clone#3      PRGSIYGQ---------------------------------
```

b

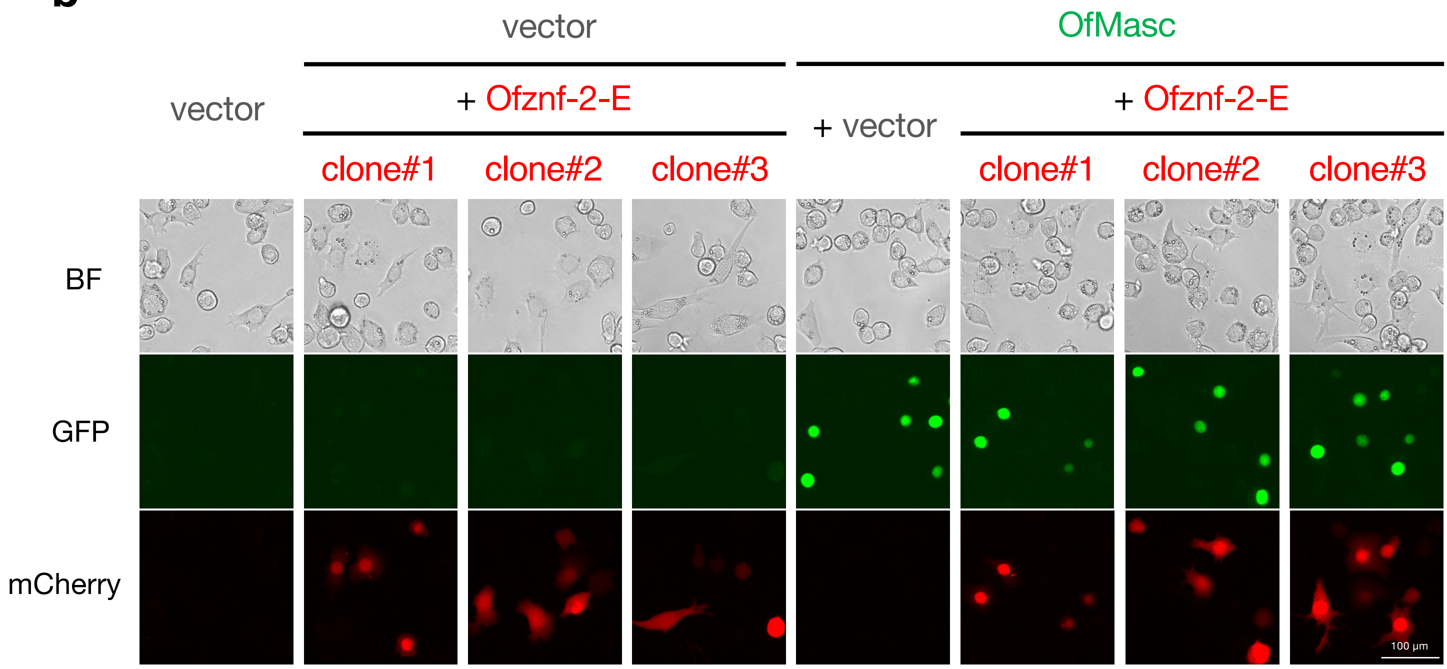

Fig. S9. Characterization of three *Ofznf-2-E* clones. (**a**) Alignment of Ofznf-2 protein sequences deduced from the version #2 genome assembly and three *Ofznf-2-E* clones isolated by RT-PCR from total RNA of egg masses at 12 hpo. (**b**) Colocalization of OfMasc and Ofznf-2-E proteins. BmN-4 cells were co-transfected with *OfMasc-GFP* and *Ofznf-2-E-mCherry* constructs. Expression and subcellular localization of GFP- and mCherry-tagged proteins were visualized via fluorescence microscopy three days post-transfection. The images of “Vector + Ofznf-2-E (clone#1)” and “OfMasc + Ofznf-2-E (clone#1)” are the same as in Fig. 3b. Similar results were obtained in two independent experiments. Source data are available in the source data file.

Fig. S10

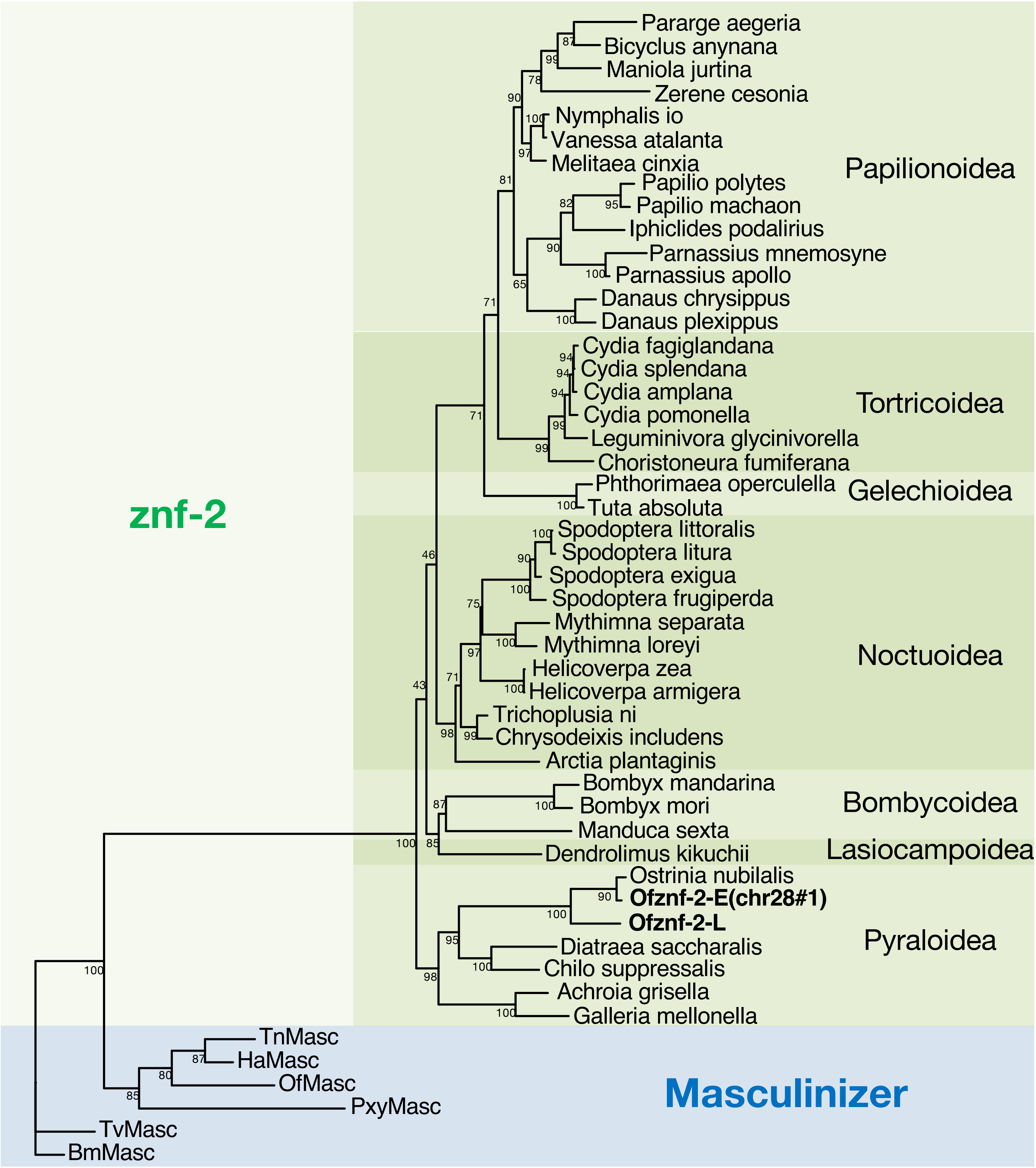

Fig. S10. Conservation of Ofznf-2 homologs across Lepidoptera. Phylogenetic analysis of znf-2 proteins in Lepidoptera. Masc proteins were included as outgroups for comparison. Protein sequence accession numbers and references are provided in Table S4. Bootstrap values for branches are indicated, reflecting the robustness of the phylogenetic relationships.

Fig. S11

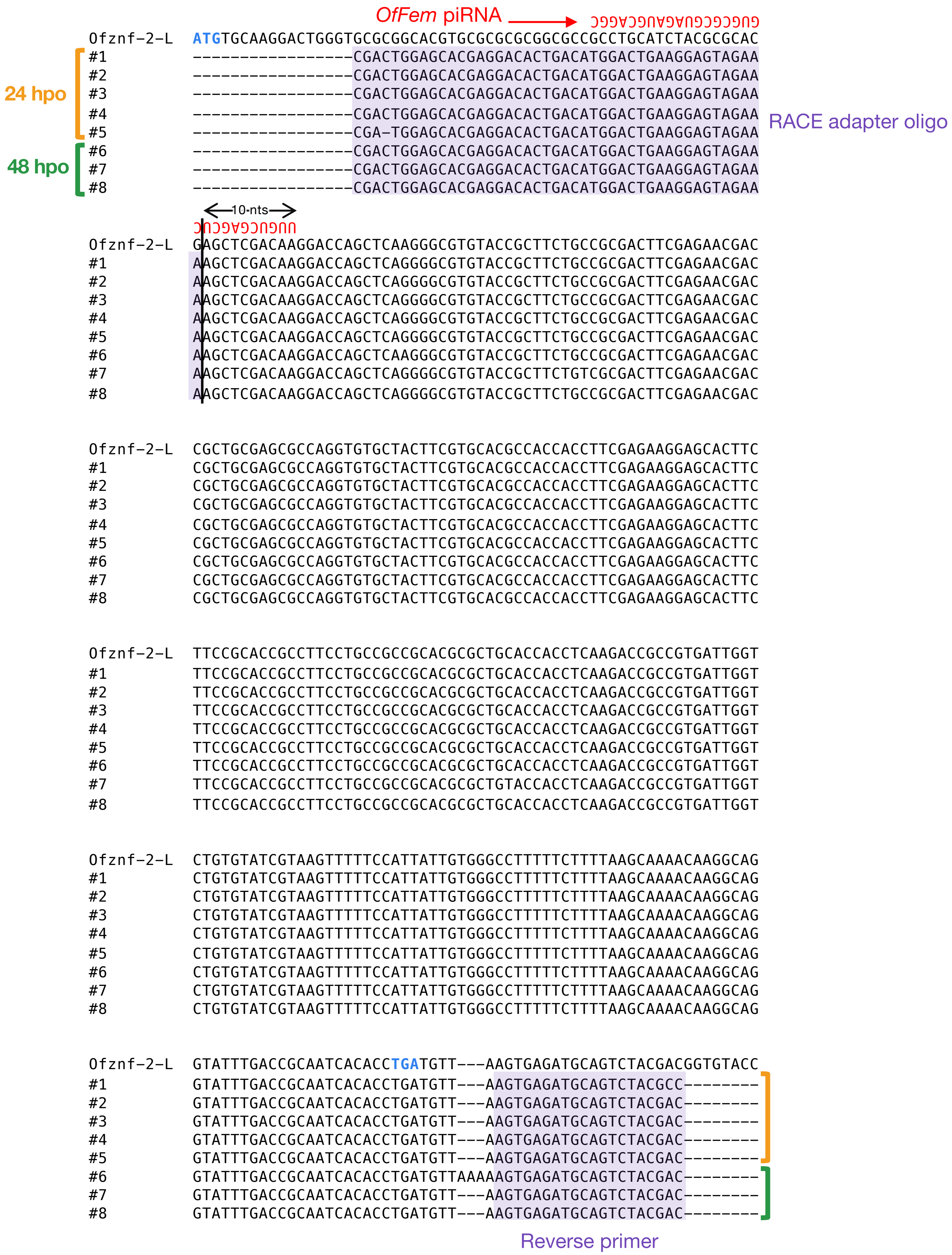

Fig. S11. Nucleotide sequences of modified RACE products for *Ofznf-2*. Modified RACE experiments were performed using total RNA from egg masses at 24 and 48 hpo. The predicted mRNA cleavage site on *Ofznf*-2 is denoted by a black line within the sequence data.

Fig. S12

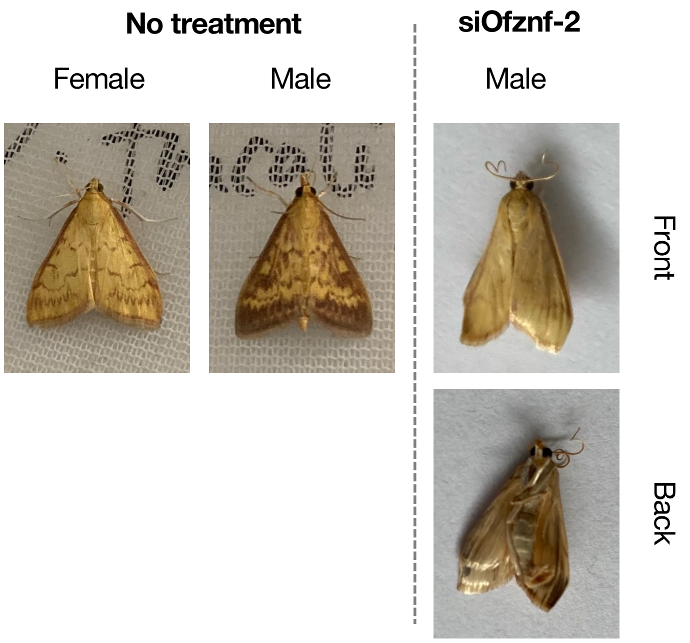

Fig. S12. Sexual mosaic phenotype in *Ostrinia furnacalis* adult females injected with siRNA targeting *Ofznf-2*. Representative images showing the sexual mosaic phenotype are presented. Source data are available in the source data file.

Fig. S13

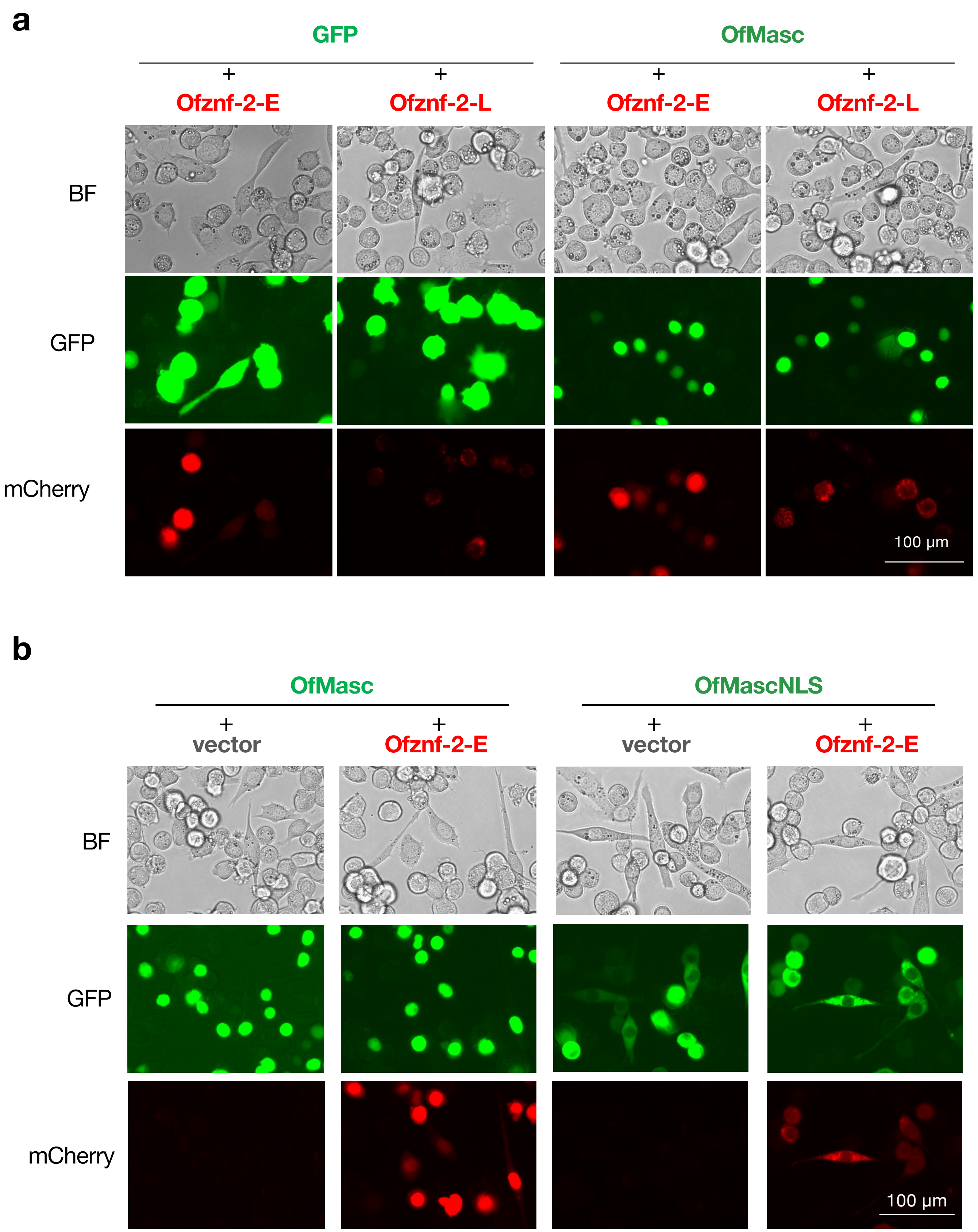

Fig. S13. Interaction between Ofznf-2-E and OfMasc proteins. BmN-4 cells were co-transfected with cDNAs encoding *GFP* or *OfMasc-GFP* and *Ofznf-2-E-mCherry* or *Ofznf-2-L-mCherry* (**a**), with cDNAs encoding *OfMasc-GFP* or *OfMascNLS-GFP* and *Ofznf-2-E-mCherry* (**b**). Fluorescence microscopy was used to visualize the subcellular localization of GFP- and mCherry-tagged proteins three days post-transfection. Similar results were obtained in two independent experiments. Source data are available in the source data file.

Fig. S14

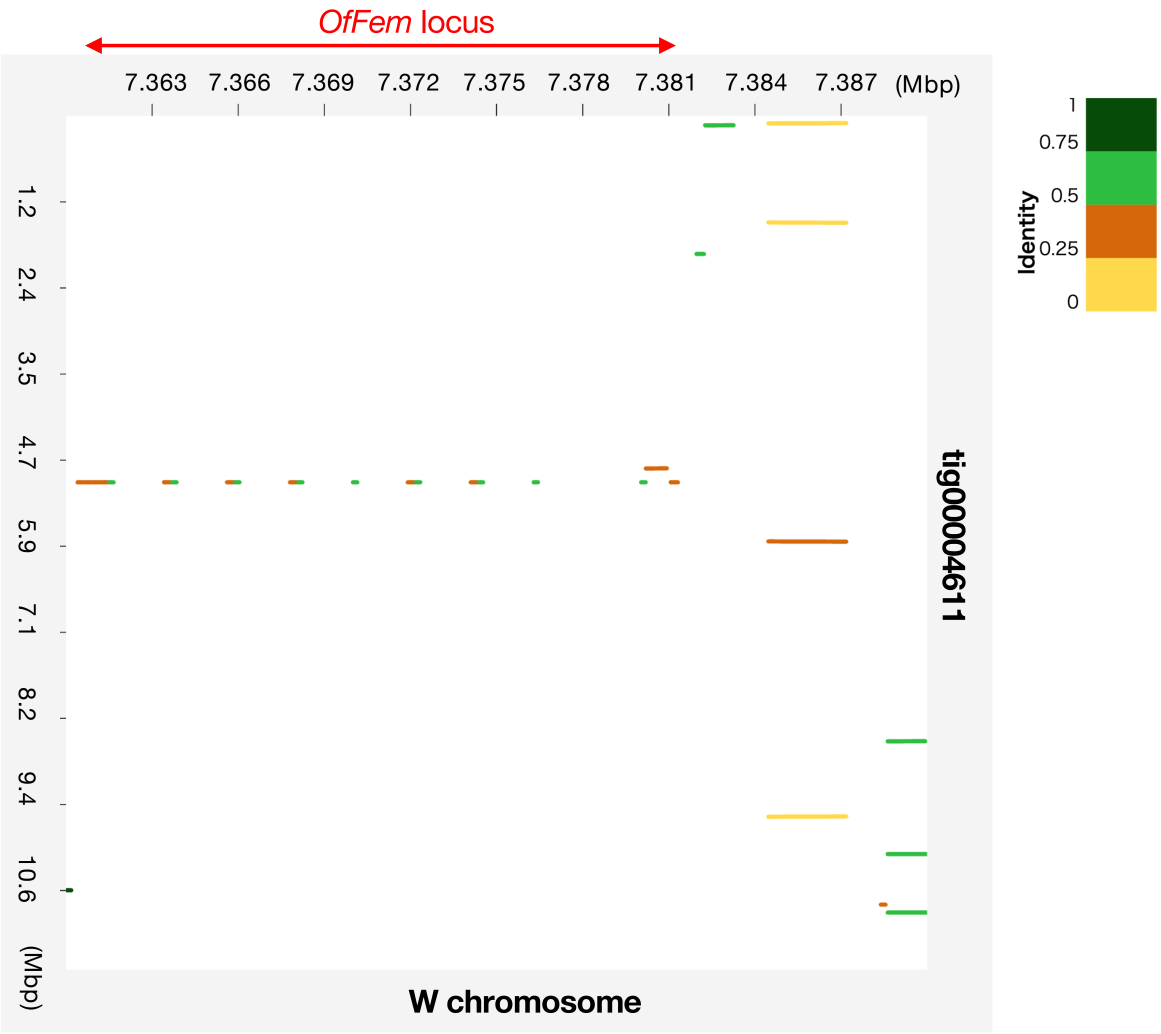

Fig. S14. Nucleotide alignment of the *OfFem* locus with a contig from the *Wolbachia*-infected *Ostrinia furnacalis* genome assembly. Dot plots comparing the *OfFem* locus and contig tig00004611 were generated using D-GENIES (*2*).

Fig. S15

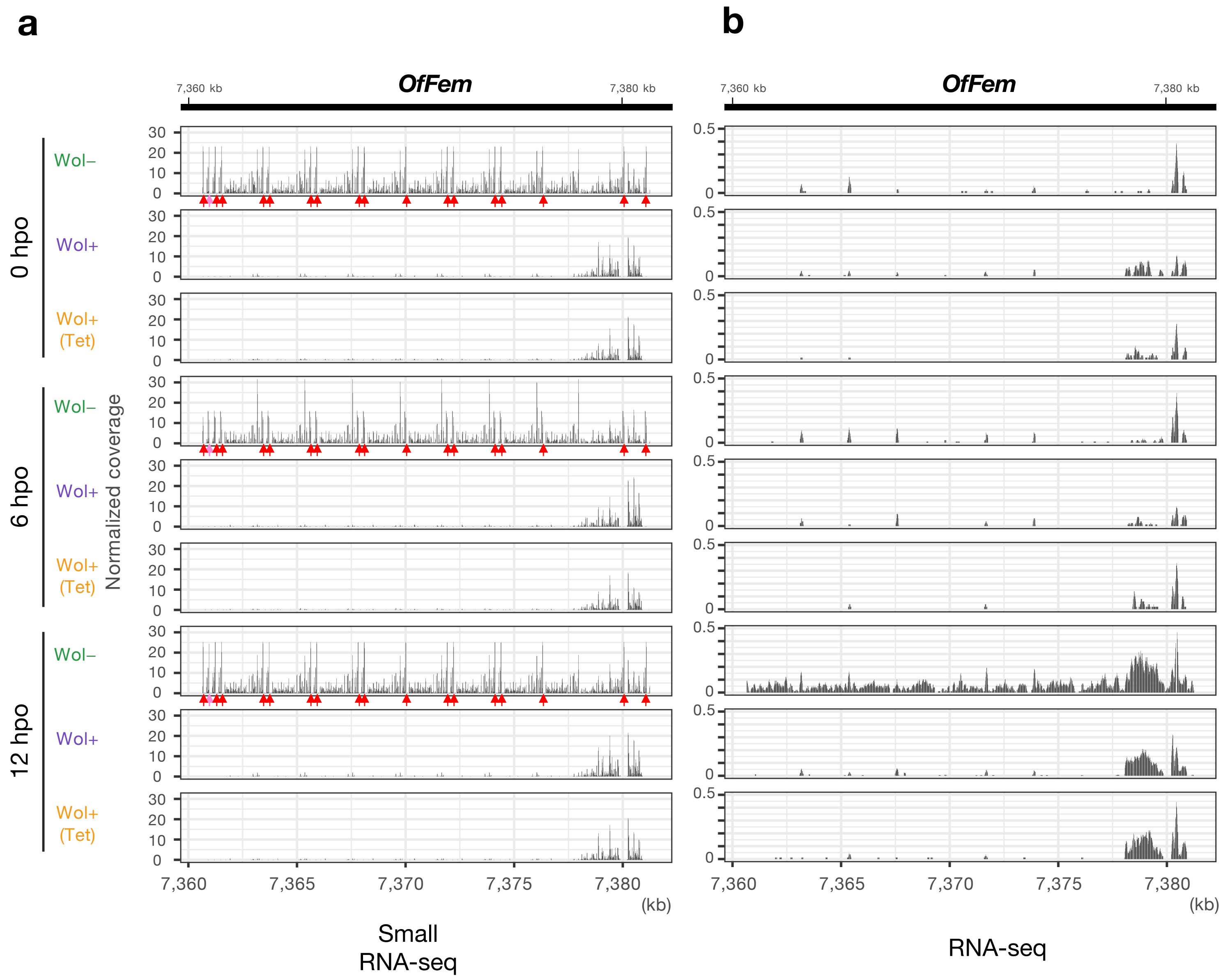

Fig. S15. Expression profiles of the *OfFem* locus and *OfFem* piRNA in the *Wolbachia*-infected *Ostrinia furnacalis* lineage. (**a**) Mapping of small RNA-seq reads from egg masses at 0, 6, and 12 hpo, to the *OfFem* locus. (**b**) Mapping of RNA-seq reads under the same conditions. Arrows indicate *OfFem* piRNA-producing sites. Red arrows denote *OfFem* piRNA reads, while pink arrows represent sequences with a single nucleotide mismatch. Wol−: uninfected; Wol+: *Wolbachia*-infected; Wol+ (Tet): tetracycline-treated *Wolbachia*-infected.

Fig. S16

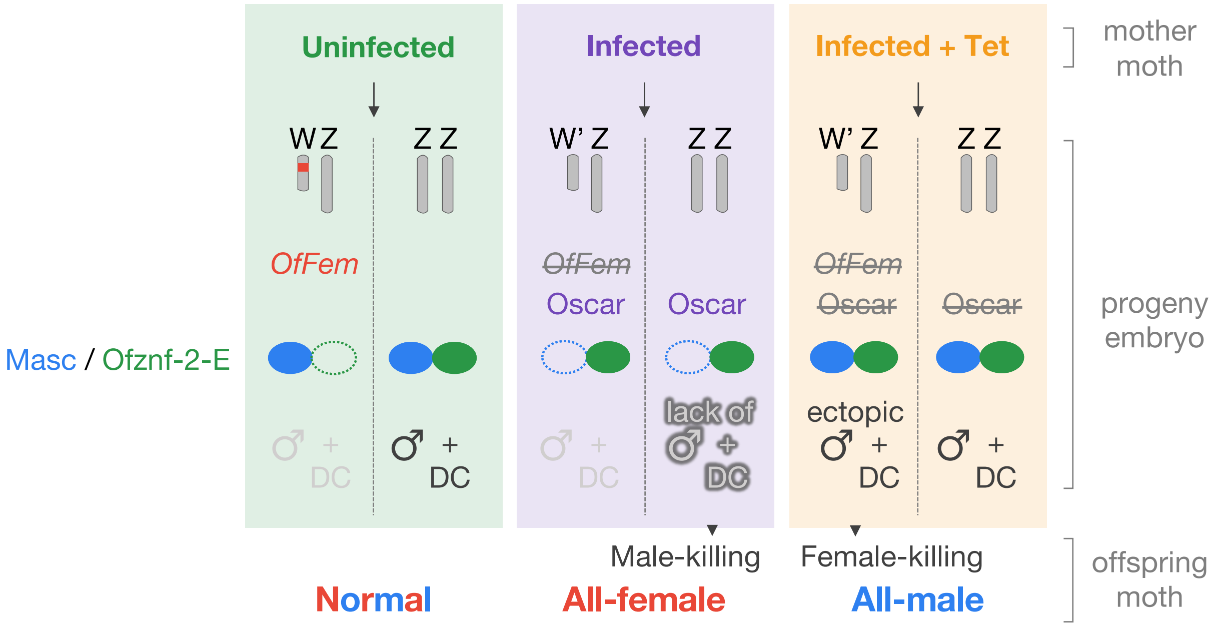

Fig. S16. Proposed mechanism for female killing in tetracycline-treated *Wolbachia*-infected moths. DC: dosage compensation; Tet: tetracycline-treatment.

Fig. S17

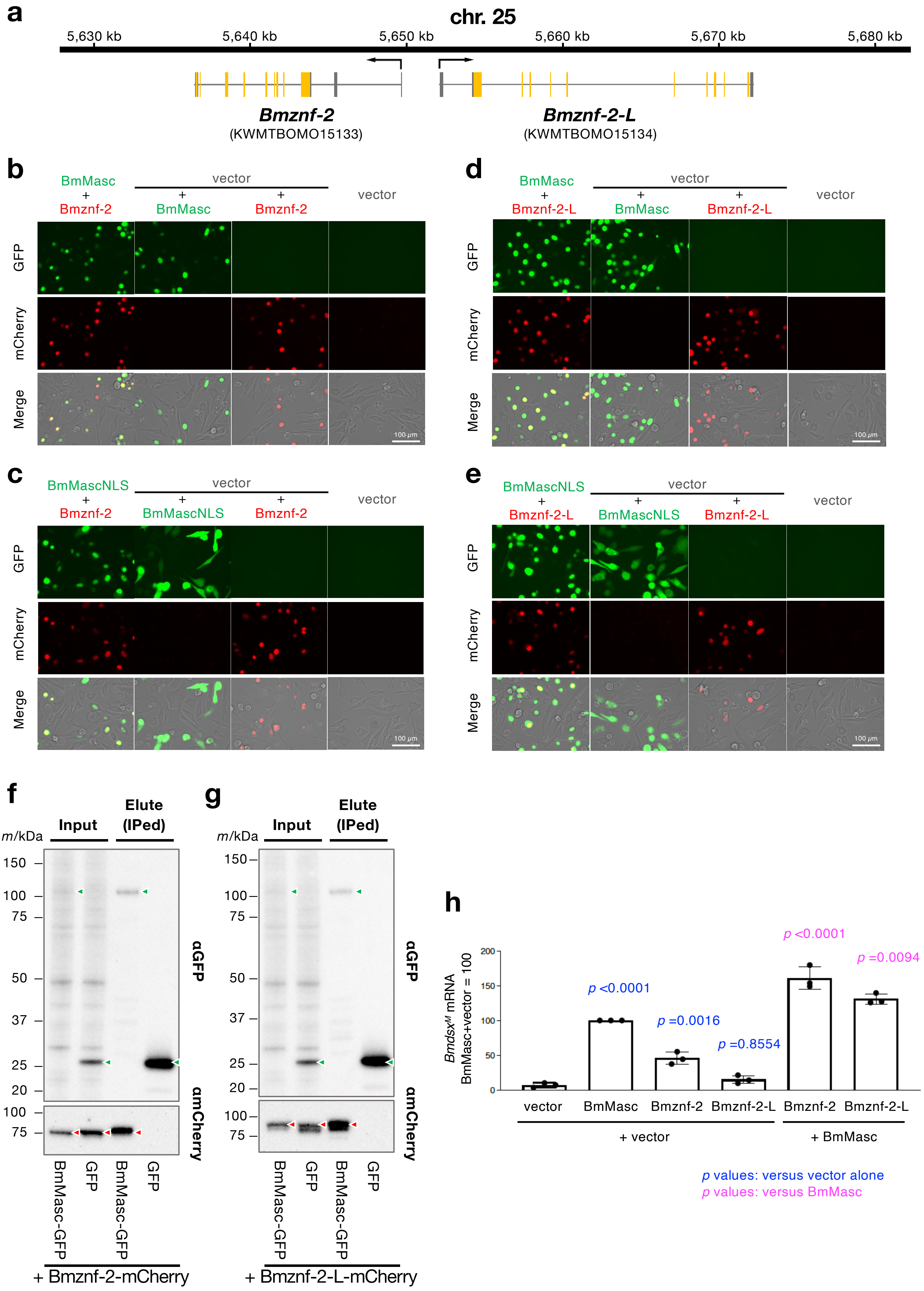

a
chr. 25
5,630 kb
5,640 kb
5,650 kb
5,660 kb
5,670 kb
5,680 kb
Bmznf-2
(KWMTBOMO15133)
Bmznf-2-L
(KWMTBOMO15134)
b
BmMasc + Bmznf-2
vector + BmMasc
vector + Bmznf-2
vector
GFP
mCherry
Merge
100 μm
c
BmMascNLS + Bmznf-2
vector + BmMascNLS
vector + Bmznf-2
vector
d
BmMasc + Bmznf-2-L
vector + BmMasc
vector + Bmznf-2-L
vector
e
BmMascNLS + Bmznf-2-L
vector + BmMascNLS
vector + Bmznf-2-L
vector
f
Input
Elute (IPed)
m/kDa
150
100
75
50
37
25
20
αGFP
αmCherry
BmMasc-GFP
GFP
BmMasc-GFP
GFP
+ Bmznf-2-mCherry
g
+ Bmznf-2-L-mCherry
h
Bmdsx^M mRNA
BmMasc+vector = 100
200
150
100
50
0
p <0.0001
p =0.0016
p =0.8554
p <0.0001
p =0.0094
vector
BmMasc
Bmznf-2
Bmznf-2-L
Bmznf-2
Bmznf-2-L
+ vector
+ BmMasc
p values: versus vector alone
p values: versus BmMasc

Fig. S17. Interaction of Bmznf-2 and Bmznf-2L with BmMasc in *Bombyx mori*. (**a**) Organization and gene structures of *Bmznf-2* and *Bmznf-2L* in the *B. mori* genome. (**b–e**) Fluorescence microscopy of BmN-4 cells co-transfected with cDNAs encoding *BmMasc-GFP* (b and d) or *BmMascNLS-GFP* (c and e), and *Bmznf-2-mCherry* (b and c) or *Bmznf-2-L-mCherry* cDNAs (d and e), or *BmMasc-GFP*, *BmMascNLS-GFP*, *Bmznf-2-mCherry*, or *Bmznf-2-L-mCherry* cDNAs alone. Similar results were obtained in two independent experiments. (**f** and **g**) Co-IP experiments. BmN-4 cells were co-transfected with *BmMasc-GFP* and *Bmznf-2-mCherry* (f) or *Bmznf-2-L-mCherry* (g) cDNAs. Immunoprecipitates obtained using anti-GFP nanobody beads were immunoblotted with an anti-mCherry antibody. Bands corresponding to GFP-tagged and mCherry-tagged proteins are marked with green and red arrowheads, respectively. Similar results were obtained in two independent experiments. (**h**) Expression of the male-type *Bmdsx* ($Bmdsx^M$) in BmN-4 cells co-transfected with cDNAs encoding *BmMascR-GFP* and *Bmznf-2-mCherry* or *Bmznf-2-L-mCherry*. Data shown are means ± SD of three independent experiments. Statistical significance was evaluated using one-way ANOVA followed by Tukey's multiple comparison tests, with adjusted *p*-values provided. Source data are available in the source data file.

Table S1. Assembly statistics of the *Ostrinia furnacalis* genomes

	version #1	version #2
Genome assembly statistics		
#Scaffolds	38	56
Total scaffold length (bp)	491,418,001	494,539,300
Longest scaffold (bp)	26,374,723	26,425,188
Contig N50 (bp)	16,166,190	16,243,591
Contig L50	14	14
Scaffold N50 (bp)	16,461,050	16,243,591
Scaffold L50	13	14
Gaps (bp)	1,400	200
BUSCO evaluation (v5.8.0, genome mode, lepidoptera_odb10, 5286genes)		
Complete BUSCOs (%)	99.3	99.3
single-copy BUSCOs (%)	99.2	99.1
duplicated BUSCOs (%)	0.1	0.1
Fragmented BUSCOs (%)	0.4	0.4
Missing BUSCOs (%)	0.3	0.4

Table S2. The positions of *Ofznf-2* copies in the *Ostrinia* genomes

Name	CDS_start	CDS_end	CDS_length	Chromosome	Reference
Ofznf-2-L	3089961	3089638	324	chr. 28	version #2
Ofznf-2-E(chr. 28#1)	6672131	6671712	420	chr. 28	version #2
Ofznf-2-E(chr. 28#2)	6764483	6764103	381	chr. 28	version #2
Ofznf-2-E(chr. Z)	163434	162871	564	chr. Z	version #2
Ofznf-2-L	2968048	2967725	324	chr. 28	version #1
Ofznf-2-E(chr. Z)	151885	151322	564	chr. Z	version #1
Ofznf-2-L	3170067	3169744	324	chr. 24*	Dai et al. (1)
Ofznf-2-E(chr. 24)	6856522	6856103	420	chr. 24*	Dai et al. (1)
Ofznf-2-E(chr. Z)	175715	175152	564	chr. Z	Dai et al. (1)

* Chr. 24 in Dai et al. (1) corresponds to chr. 28 in version #1 and version #2.

Table S3. Oligonucleotides used in this study

Class	Name	Sequence (5'-3')	Reference
RT-PCR	Ofdsx-1F	CATACACCCGACAGGAGTTGAATTG	Sugimoto et al. (3)
RT-PCR	Ofdsx-5R	CAGCACATCGAGTACGAGGAGC	Sugimoto et al. (3)
RT-PCR	Kasumi-both_1F	CTACTTCGTGCACGCCACCACCTTC	This study
RT-PCR	Kasumi-both_1R	TCGCGTACCACTTAAGGTTTGCAGTT	This study
RT-qPCR	Kasumi-F	CCATGTGCAAGGACTGGGT	This study
RT-qPCR	Kasumi-R	TGCACGAAGTAGCACACCTG	This study
RT-qPCR	Kasumi-E-F	AAGGAGCACTTCTTCCGCAC	This study
RT-qPCR	Kasumi-E-R	AGACCAATCACTAGTTCGCAGAT	This study
genomic PCR, RT-PCR	OfFem-F	TGCATCTACGCGCACGAGCT	This study
genomic PCR, RT-PCR	OfFem-R	CTCCACCTCCTGCTTCAGCGTGAG	This study
Modified RACE	Kasumi-X1-R1	GTCGTAGACTGCATCTCACT	This study
siRNA	siKasumi-2-gui	AAGUGCUCCUUCUCGAAGGUG	This study
siRNA	siKasumi-2-pas	CCUUCGAGAAGGAGCACUUUU	This study
RT-qPCR	OfdsxM-F2	GAAGATTGATGAAGCCCACTG	Katsuma et al. (4)
RT-qPCR	OfdsxM-R1	GCACTGTGTCTATCACACTG	Katsuma et al. (4)
RT-qPCR	Ofdsx-common-F	GTGGTCAAGGCACCCAGAAG	This study
RT-qPCR	Ofdsx-common-R	CATCATCTCCCAGGAATAGTGGAA	This study
RT-qPCR	Bmrp49-F	CCCAACATTGGTTACGGTTC	This study
RT-qPCR	Bmrp49-R	GCTCTTTCCACGA TCAGCTT	This study
RT-qPCR	BmdsxM-qF1a	CAAGGAAAATCTACGAAGGTTA	This study
RT-qPCR	BmdsxM-qR1	GGTCATGCGCCGTCTGTATC	This study

Table S4. Amino acid sequences used in the phylogenetic analysis

Class	Label	Accession/reference
Masculinizer	BmMasc	BAO79517.1
Masculinizer	TvMasc	BAS02075.1
Masculinizer	OfMasc	BAS02074.1
Masculinizer	PxyMasc	Harvey-Samuel et al. (5)
Masculinizer	HaMasc	QCD63870.1
Masculinizer	HcMasc	Li et al., 2023
Masculinizer	CpMasc	MW505945.1
Masculinizer	BaMasc	WIG62490.1
znf-2	Bmznf-2	Kawamoto et al. (6)
znf-2	Bmznf-2-like	Kawamoto et al. (6)
znf-2	*Ostrinia nubilalis*	XP_063837912.1
znf-2	*Galleria mellonella*	XP_052751489.1
znf-2	*Achroia grisella*	XP_059055227.1
znf-2	*Chilo suppressalis*	CAH0684234.1
znf-2	*Mythimna loreyi*	KAJ8737853.1
znf-2	*Mythimna separata*	KAJ8736944.1
znf-2	*Diatraea saccharalis*	CAG9781691.1
znf-2	*Chrysodeixis includens*	CAH0577956.1
znf-2	*Arctia plantaginis*	CAB3245850.1
znf-2	*Trichoplusia ni*	XP_026739720.1
znf-2	*Manduca sexta*	KAG6462998.1
znf-2	*Spodoptera litura*	XP_022833137.1
znf-2	*Spodoptera littoralis*	CAB3517592.1
znf-2	*Spodoptera frugiperda*	KAF9799242.1
znf-2	*Spodoptera exigua*	KAF9421981.1
znf-2	*Bombyx mori*	XP_004924546.1
znf-2	*Bombyx mandarina*	XP_028033799.1
znf-2	*Dendrolimus kikuchii*	KAJ0184093.1
znf-2	*Helicoverpa armigera*	XP_021193997.3
znf-2	*Helicoverpa zea*	XP_047040617.1
znf-2	*Choristoneura fumiferana*	KAI8431072.1
znf-2	*Danaus plexippus*	OWR43715.1
znf-2	*Cydia pomonella*	XP_061722065.1
znf-2	*Cydia splendana*	XP_063633078.1
znf-2	*Cydia fagiglandana*	XP_063394536.1
znf-2	*Cydia amplana*	XP_063372791.1
znf-2	*Bicyclus anynana*	XP_052746431.1
znf-2	*Leguminivora glycinivorell*	XP_048006289.1
znf-2	*Papilio machaon*	XP_014366263.1
znf-2	*Maniola jurtina*	XP_045784521.1
znf-2	*Parnassius apollo*	CAG5039184.1
znf-2	*Parnassius mnemosyne*	CAK1588573.1
znf-2	*Zerene cesonia*	XP_038219946.1
znf-2	*Papilio polytes*	XP_013146496.1
znf-2	*Pararge aegeria*	XP_039763743.1
znf-2	*Melitaea cinxia*	XP_045458137.1
znf-2	*Vanessa atalanta*	XP_047544155.1
znf-2	*Tuta absoluta*	KAJ2953993.1
znf-2	*Phthorimaea operculella*	KAI5640386.1
znf-2	*Iphiclides podalirius*	CAH2034868.1
znf-2	*Danaus chrysippus*	CAG9558112.1
znf-2	*Nymphalis io*	XP_050361024.1

Table S5. Chromosome numbers in *O. furnacalis* genome assemblies used in this study

Assembly	Chromosome numbers in *B. mori* (Kawamoto et al. (6))																															Reference
	1 (Z)	2	3	4	5	6	7	8	9	10	11		12	13	14	15	16	17	18	19	20	21	22	23		24		25	26	27	28	
version #1/#2	1 (Z)	2	3	4	5	6	7	8	9	10	11a	11b	12	13	14	15	16	17	18	19	20	21	22	23a	23b	24a	24b	25	26	27	28	This study
Dai et al. (1)	Z	30	16	5	2	11	20	10	8	6	28	13	1	7	19	3	21	9	14	15	23	17	4	12	27	26	29	18	22	25	24	GCA_038426615.1; Dai et al. (1)

Note: Dai et al. (1) and GCA_038426615.1 referred to the same genome assembly, but they used different chromosome numbering systems.
(e.g., chromosome 28 in GCA_038426615.1 corresponded to linkage group 26 in Dai et al. (1))
In this study, we followed the chromosome numbering used in GCA_038426615.1.

Supplementary references

1. W. Dai, J. E. Mank, L. Ban, Gene gain and loss from the Asian corn borer W chromosome. *BMC Biol.* **22**, 102 (2024).
2. F. Cabanettes, C. Klopp, D-GENIES: dot plot large genomes in an interactive, efficient and simple way. *Peer J* **6**, e4958 (2018).
3. T. N. Sugimoto, Y. Ishikawa, A male-killing *Wolbachia* carries a feminizing factor and is associated with degradation of the sex-determining system of its host. *Biol. Lett*. **8**, 412–415 (2012).
4. S. Katsuma, K. Hirota, N. Matsuda-Imai, T. Fukui, T. Muro, K. Nishino, H. Kosako, K. Shoji, H. Takanashi, T. Fujii, S-i. Arimura, T. Kiuchi, A *Wolbachia* factor for male killing in lepidopteran insects. *Nat. Commun*. **13**, 1–12 (2022).
5. T. Harvey-Samuel, V. C. Norman, R. Carter, E. Lovett, L. Alphey, Identification and characterization of a *Masculinizer* homologue in the diamondback moth, *Plutella xylostella*. *Insect Mol. Biol.* **29**, 231–240 (2020).
6. M. Kawamoto, A. Jouraku, A. Toyoda, K. Yokoi, Y. Minakuchi, S. Katsuma, A. Fujiyama, T. Kiuchi, K. Yamamoto, T. Shimada, High-quality genome assembly of the silkworm, *Bombyx mori*. *Insect Biochem. Mol. Biol.* **107**, 53–62 (2019).
