# Supplementary material for: Complete transition from chromosomal to cytoplasmic sex determination during prolonged Wolbachia symbiosis
Source: Nat Commun. 2026 Jan 8;17:104. doi: 10.1038/s41467-025-67993-x (PMC12783822; doi:10.1038/s41467-025-67993-x)
Supplement: Supplementary file 2 — Description of Additional Supplementary File [file 41467_2025_67993_MOESM2_ESM.pdf]

Description of Additional Supplementary File

Supplementary Data 1 –
Accession number for each chromosome in *O. furnacalis* genome assemblies used in this study.
