# Supplementary material for: Complete transition from chromosomal to cytoplasmic sex determination during prolonged Wolbachia symbiosis
Source: Nat Commun. 2026 Jan 8;17:104. doi: 10.1038/s41467-025-67993-x (PMC12783822; doi:10.1038/s41467-025-67993-x)
Supplement: Supplementary file 4 — Reporting Summary [file 41467_2025_67993_MOESM4_ESM.pdf]

nature portfolio

Corresponding author(s): Susumu Katsuma

Last updated by author(s): Oct 28, 2025

Reporting Summary

Nature Portfolio wishes to improve the reproducibility of the work that we publish. This form provides structure for consistency and transparency in reporting. For further information on Nature Portfolio policies, see our Editorial Policies and the Editorial Policy Checklist.

Statistics

For all statistical analyses, confirm that the following items are present in the figure legend, table legend, main text, or Methods section.

n/a	Confirmed	
☐	☒	The exact sample size (*n*) for each experimental group/condition, given as a discrete number and unit of measurement
☐	☒	A statement on whether measurements were taken from distinct samples or whether the same sample was measured repeatedly
☐	☒	The statistical test(s) used AND whether they are one- or two-sided<br>*Only common tests should be described solely by name; describe more complex techniques in the Methods section.*
☒	☐	A description of all covariates tested
☐	☒	A description of any assumptions or corrections, such as tests of normality and adjustment for multiple comparisons
☐	☒	A full description of the statistical parameters including central tendency (e.g. means) or other basic estimates (e.g. regression coefficient) AND variation (e.g. standard deviation) or associated estimates of uncertainty (e.g. confidence intervals)
☐	☒	For null hypothesis testing, the test statistic (e.g. *F*, *t*, *r*) with confidence intervals, effect sizes, degrees of freedom and *P* value noted<br>*Give P values as exact values whenever suitable.*
☒	☐	For Bayesian analysis, information on the choice of priors and Markov chain Monte Carlo settings
☒	☐	For hierarchical and complex designs, identification of the appropriate level for tests and full reporting of outcomes
☒	☐	Estimates of effect sizes (e.g. Cohen's *d*, Pearson's *r*), indicating how they were calculated

Our web collection on statistics for biologists contains articles on many of the points above.

Software and code

Policy information about availability of computer code

Data collection	Image Lab 6.0 (Bio-Rad), FLoid Cell Imaging station(Life Technologies), StepOne Software v2.3 (Thermo Fisher Scientific)
Data analysis	Proteome Discoverer version 2.5 (Thermo Fisher Scientific), Canu v.2.1.1, BWA v.0.7.8, v.0.7.17, Pilon v.1.23, PGAP v.2021-05-19.build5429, ClustalW v.2.1, IQ-TREE v.2.2.0.3, trimAl v.1.4.1, BLASTn v.2.7.1, v2.9.0, v.2.12.0, tBLASTn v.2.12.0, BLASTp v.2.9.0, Skyline v.20.2, Prism 9 (GraphPad), Image Lab 6.0 (Bio-Rad), SMART (a Simple Modular Architecture Research Tool, http://smart.embl-heidelberg.de), StepOne Software v2.3 (Thermo Fisher Scientific), Trinity v.2.15.1, Salmon v.1.10.2, Wasabi v.1.0.1, Sleuth v.0.30.1, SAMtools v.1.17, BEDTools v.2.31.0, HISAT2 v.2.2.1, Bowtie v.1.3.1, Subread v.2.0.6, edgeR v.3.42.4, RepeatModeler v.2.0.4, Pilon v1.4.0, Purge_dups v1.2.5, YaHS v1.2a, HiLine v0.2.4, Juicebox v2.16.00

For manuscripts utilizing custom algorithms or software that are central to the research but not yet described in published literature, software must be made available to editors and reviewers. We strongly encourage code deposition in a community repository (e.g. GitHub). See the Nature Portfolio guidelines for submitting code & software for further information.

Data

Policy information about availability of data

All manuscripts must include a data availability statement. This statement should provide the following information, where applicable:

- Accession codes, unique identifiers, or web links for publicly available datasets
- A description of any restrictions on data availability
- For clinical datasets or third party data, please ensure that the statement adheres to our policy

The public data used are as follows: O. furnacalis protein data (GCF_004193835.1_ASM419383v1_protein.faa [https://ftp.ncbi.nlm.nih.gov/genomes/all/GCF/004/193/835/GCF_004193835.1_ASM419383v1/]). The genome sequences of O. furnacalis have been deposited in DNA Data Bank of Japan under accession numbers AP039452-AP039489 (version #1) and AP039490-AP039545 (version #2). Raw sequencing data have been deposited in GenBank under accession numbers DRR462151–DRR462186, DRR629067–DRR629078, DRR632127–DRR632132 (RNA-seq), DRR623824–DRR623829, DRR408740–DRR408744, DRR626685–DRR626691 (small RNA-seq), PRJDB14515, SRR31936107, SRR31936108, and SRR31944223 (genome). The MS proteomics data have been deposited to the ProteomeXchange Consortium via the jPOST partner repository with the dataset identifier PXD059481.

Research involving human participants, their data, or biological material

Policy information about studies with human participants or human data. See also policy information about sex, gender (identity/presentation), and sexual orientation and race, ethnicity and racism.

Reporting on sex and gender	*Use the terms sex (biological attribute) and gender (shaped by social and cultural circumstances) carefully in order to avoid confusing both terms. Indicate if findings apply to only one sex or gender; describe whether sex and gender were considered in study design; whether sex and/or gender was determined based on self-reporting or assigned and methods used. Provide in the source data disaggregated sex and gender data, where this information has been collected, and if consent has been obtained for sharing of individual-level data; provide overall numbers in this Reporting Summary. Please state if this information has not been collected. Report sex- and gender-based analyses where performed, justify reasons for lack of sex- and gender-based analysis.*
Reporting on race, ethnicity, or other socially relevant groupings	*Please specify the socially constructed or socially relevant categorization variable(s) used in your manuscript and explain why they were used. Please note that such variables should not be used as proxies for other socially constructed/relevant variables (for example, race or ethnicity should not be used as a proxy for socioeconomic status). Provide clear definitions of the relevant terms used, how they were provided (by the participants/respondents, the researchers, or third parties), and the method(s) used to classify people into the different categories (e.g. self-report, census or administrative data, social media data, etc.) Please provide details about how you controlled for confounding variables in your analyses.*
Population characteristics	*Describe the covariate-relevant population characteristics of the human research participants (e.g. age, genotypic information, past and current diagnosis and treatment categories). If you filled out the behavioural & social sciences study design questions and have nothing to add here, write "See above."*
Recruitment	*Describe how participants were recruited. Outline any potential self-selection bias or other biases that may be present and how these are likely to impact results.*
Ethics oversight	*Identify the organization(s) that approved the study protocol.*

Note that full information on the approval of the study protocol must also be provided in the manuscript.

Field-specific reporting

Please select the one below that is the best fit for your research. If you are not sure, read the appropriate sections before making your selection.

☒ Life sciences ☐ Behavioural & social sciences ☐ Ecological, evolutionary & environmental sciences

For a reference copy of the document with all sections, see nature.com/documents/nr-reporting-summary-flat.pdf

Life sciences study design

All studies must disclose on these points even when the disclosure is negative.

Sample size	Sample sizes were selected based on the data obtained in previous studies.
Data exclusions	No data were excluded.
Replication	Experiments were repeated or replicated as described in the figure legends. All experiments shown could be reproduced as described.
Randomization	No randomization was performed. Experiments were performed by treating samples and controls side by side under indicated conditions.

Blinding	Egg sexes are not visually distinguished. For other experiments, blinding was not performed. Wherever possible, unbiased experimental procedures and data analysis were performed.

Reporting for specific materials, systems and methods

We require information from authors about some types of materials, experimental systems and methods used in many studies. Here, indicate whether each material, system or method listed is relevant to your study. If you are not sure if a list item applies to your research, read the appropriate section before selecting a response.

Materials & experimental systems

n/a	Involved in the study
☐	☒ Antibodies
☐	☒ Eukaryotic cell lines
☒	☐ Palaeontology and archaeology
☐	☒ Animals and other organisms
☒	☐ Clinical data
☒	☐ Dual use research of concern
☒	☐ Plants

Methods

n/a	Involved in the study
☒	☐ ChIP-seq
☒	☐ Flow cytometry
☒	☐ MRI-based neuroimaging

Antibodies

Antibodies used	Primary antibodies:<br>anti-GFP antibody (MBL, 598: 1:5000 dilution)<br>anti-RFP antibody (MBL, PM005: 1:5000 dilution)<br><br>Secondary antibodies:<br>Goat F(ab')2 Anti-Rabbit IgG - H&L (AP), pre-adsorbed (Abcam, ab 98505: 1:10000 dilution)
Validation	anti-GFP and anti -RFP antibodies are produced in rabbit and validated by MBL.

Eukaryotic cell lines

Policy information about cell lines and Sex and Gender in Research

Cell line source(s)	BmN-4 (Bombyx mori, provided by C. Yasunaga-Aoki), OfT1C (in house, Ostrinia furnacalis). BmN-4 is available from NBRP Silkworm (https://shigen.nig.ac.jp/silkwormbase/cultured_cells.jsp).
Authentication	Every cell line was authenticated by sequencing several genes.
Mycoplasma contamination	Not checked.
Commonly misidentified lines<br>(See ICLAC register)	No commonly misidentified cell lines were used in the study.

Animals and other research organisms

Policy information about studies involving animals; ARRIVE guidelines recommended for reporting animal research, and Sex and Gender in Research

Laboratory animals	*For laboratory animals, report species, strain and age OR state that the study did not involve laboratory animals.*
Wild animals	Ostrinia furnacalis (corn borer moths), O. scapulalis, female and male, all developmental stages, collected at Nishi-Tokyo, Japan (35.7º N, 139.5º E) and Matsudo, Japan (35.8º N, 139.9º E), and cultured in our lab until their family lines fail.
Reporting on sex	*Indicate if findings apply to only one sex; describe whether sex was considered in study design, methods used for assigning sex. Provide data disaggregated for sex where this information has been collected in the source data as appropriate; provide overall numbers in this Reporting Summary. Please state if this information has not been collected. Report sex-based analyses where performed, justify reasons for lack of sex-based analysis.*
Field-collected samples	O. furnacalis and O. scapulalis moths used in this study were collected at Nishi-Tokyo, Japan (35.7º N, 139.5º E) in early summer of 2020 and 2021. Wolbachia-infected O. furnacalis moths were collected at Matsudo, Japan (35.8º N, 139.9º E) in early summer of 2014 and at Nishi-Tokyo, Japan (35.7º N, 139.5º E) in early summer of 2021, and Wolbachia-infected O. scapulalis moths were collected at Matsudo, Japan (35.8º N, 139.9º E) in early summer of 2020. Larval O. furnacalis and O. scapulalis were reared on an artificial diet (Insecta LFS, Nosan Corp., Japan) at 25ºC under a photoperiod of 16L and 8D in the lab.

Ethics oversight	This study does not require the ethical approval.

Note that full information on the approval of the study protocol must also be provided in the manuscript.

Plants

Seed stocks	*Report on the source of all seed stocks or other plant material used. If applicable, state the seed stock centre and catalogue number. If plant specimens were collected from the field, describe the collection location, date and sampling procedures.*
Novel plant genotypes	*Describe the methods by which all novel plant genotypes were produced. This includes those generated by transgenic approaches, gene editing, chemical/radiation-based mutagenesis and hybridization. For transgenic lines, describe the transformation method, the number of independent lines analyzed and the generation upon which experiments were performed. For gene-edited lines, describe the editor used, the endogenous sequence targeted for editing, the targeting guide RNA sequence (if applicable) and how the editor was applied.*
Authentication	*Describe any authentication procedures for each seed stock used or novel genotype generated. Describe any experiments used to assess the effect of a mutation and, where applicable, how potential secondary effects (e.g. second site T-DNA insertions, mosiacism, off-target gene editing) were examined.*
